# Epitope Predictions Indicate the Presence of Two Distinct Types of Epitope-Antibody-Reactivities Determined by Epitope Profiling of Intravenous Immunoglobulins

**DOI:** 10.1371/journal.pone.0078605

**Published:** 2013-11-11

**Authors:** Mitja Luštrek, Peter Lorenz, Michael Kreutzer, Zilliang Qian, Felix Steinbeck, Di Wu, Nadine Born, Bjoern Ziems, Michael Hecker, Miri Blank, Yehuda Shoenfeld, Zhiwei Cao, Michael O. Glocker, Yixue Li, Georg Fuellen, Hans-Jürgen Thiesen

**Affiliations:** 1 Institute for Biostatistics and Informatics in Medicine and Ageing Research, Universitätsmedizin, University of Rostock, Rostock, Germany; 2 Department of Intelligent Systems, Jožef Stefan Institute, Ljubljana, Slovenia; 3 Institute of Immunology, Universitätsmedizin Rostock, University of Rostock, Rostock, Germany; 4 Shanghai Institute for Biological Sciences, Shanghai, China; 5 Gesellschaft für Individualisierte Medizin GmbH, Rostock, Germany; 6 Steinbeis Transfer Center for Proteome Analysis, Rostock, Germany; 7 The Zabludovicz Center for Autoimmune Diseases, Sheba Medical Center, Ramat-Gan, Israel; 8 School of Life Sciences and Technology, Tongji University, Shanghai, China; 9 Proteome Center Rostock, University of Rostock, Rostock, Germany; University of Catania, Italy

## Abstract

Epitope-antibody-reactivities (EAR) of intravenous immunoglobulins (IVIGs) determined for 75,534 peptides by microarray analysis demonstrate that roughly 9% of peptides derived from 870 different human protein sequences react with antibodies present in IVIG. Computational prediction of linear B cell epitopes was conducted using machine learning with an ensemble of classifiers in combination with position weight matrix (PWM) analysis. Machine learning slightly outperformed PWM with area under the curve (AUC) of 0.884 vs. 0.849. Two different types of epitope-antibody recognition-modes (Type I EAR and Type II EAR) were found. Peptides of Type I EAR are high in tyrosine, tryptophan and phenylalanine, and low in asparagine, glutamine and glutamic acid residues, whereas for peptides of Type II EAR it is the other way around. Representative crystal structures present in the Protein Data Bank (PDB) of Type I EAR are PDB 1TZI and PDB 2DD8, while PDB 2FD6 and 2J4W are typical for Type II EAR. Type I EAR peptides share predicted propensities for being presented by MHC class I and class II complexes. The latter interaction possibly favors T cell-dependent antibody responses including IgG class switching. Peptides of Type II EAR are predicted not to be preferentially presented by MHC complexes, thus implying the involvement of T cell-independent IgG class switch mechanisms. The high extent of IgG immunoglobulin reactivity with human peptides implies that circulating IgG molecules are prone to bind to human protein/peptide structures under non-pathological, non-inflammatory conditions. A webserver for predicting EAR of peptide sequences is available at www.sysmed-immun.eu/EAR.

## Introduction

The human immune system consists of an innate and an adaptive branch. The latter encompasses B cell driven antibody-mediated humoral and T cell driven cellular immune responses. Both types of adaptive immune responses are highly connected with each other by the involvement of MHC class I and MHC class II peptide complexes (for review see [1]). In short, MHC class I complexes are found on all nucleated cells presenting intracellularly derived peptides to cytotoxic CD8-positive T cells. MHC class II complexes are found on professional antigen-presenting cells such as dendritic cells, macrophages and B cells, presenting peptides derived from extracellular uptake of proteins to CD4-positive T cells [2]. For instance, the B cell receptor (membrane bound immunoglobulin) binds antigenic (protein) structures, these complexes are then engulfed, bound proteins are dissected into peptides, which are finally presented by the MHC class II complex to the T cell receptor of CD4-positive T cells [3]. These interactions are instrumental in making the decision whether and what type of immune responses (T cell mediated cellular versus B cell mediated humoral response) are going to be promoted. In case of humoral immune responses, the T and B cells express co-stimulatory signals along with cytokines, driving forward the antigen- as well as immune complex-driven differentiation and maturation of B cells into antibody-secreting plasma cells [4]. On B cells, the antigen/epitope binding site designated paratope is located at each tip of the two Fab fragments that are displayed by membrane-bound monomeric IgM constituting the B cell receptor [5]. Once the antigen-decorated B cell gains T cell help, the B cell might be induced to perform a immunoglobulin (Ig) class switch to IgG synthesis [6]. Class switching is partly supported by the synergy of B cell receptor crosslinking and nucleic acid/immune complex-driven engagement of the Toll-like-receptor system. These processes either lead to T cell-dependent or T cell-independent antibody responses [7],[8],[9].

### Epitope Analysis

Two types of epitopes i. continuous and ii. discontinuous epitopes participate in epitope-antibody-reactivities (EAR). B cell epitopes are most commonly discontinuous (also called conformational or assembled), consisting of segments of multiple chains brought together by the folding of the protein (antigen) [10]. Only about 10% of all epitopes recognized by antibodies are thought to be continuous (also called linear or sequential) [11]. Whole protein arrays [12] generally detect both types of epitopes of an antigen within one single measure. Whereas the exact nature of a discontinuous epitope has usually to be specified by sophisticated X-ray crystallography [13], peptides displayed on microarrays are experimentally used to determine continuous epitopes [14] [15]. This is why most work on epitope profiling is focused on continuous epitopes.

### Epitope Prediction

The first attempts to predict continuous B cell epitopes were based on propensity scales [16]. Current state-of-the-art epitope prediction generally uses machine learning approaches. Larsen et al. [17] trained a Hidden Markov Model (HMM) on epitopes in conjunction with random amino acid sequences. The antigenicity of amino acids was then derived from the ratios of their emission probabilities by the epitope HMM and the random HMM. The area under curve (AUC) of receiver operating characteristic (ROC) of such a classifier in combination with a hydrophobicity scale was 0.671. Söllner and Mayer [18] used decision tree and nearest neighbor machine learning algorithms. They utilized over 1,000 attributes related to relative positions of amino acids in the sequences and over 250 propensity scales. The post test probability (a measure similar to accuracy) of the best classifier was 69.31%. In the studies cited below, the classifiers used have been trained and tested on B cell epitopes from the Bcipep database [19] as positive examples and random peptides as negative examples (except for Rubinstein et al. [13], who apparently used epitope and non-epitope parts of the same antigens as positive and negative examples). Saha and Raghava [20] employed a recurrent neural network. The accuracy of the trained network was 65.93%. Chen et al. [21] used the support vector machine learning algorithm. Their attributes were frequencies of amino acid pairs and five propensity scores. The resulting accuracy was 73.71%. El-Manzalawy et al. [22], [23] used support vector machines in combination with a subsequence kernel. Their attributes were all subsequences of peptides up to a certain length, including subsequences with gaps. The AUC of the subsequence kernel classification was 0.812 and the accuracy 73.37% ([23], original data set). They reimplemented Chen et al.’s [21] method, which yielded an AUC of 0.717 and an accuracy of 65.65%. The difference compared to the accuracy reported by Chen et al. is probably due to differences in the exact composition of the data set. Results reported by El-Manzalawy et al. confirm the superiority of the subsequence kernel classification. Rubinstein et al. [13] used the naive Bayes machine learning algorithm. Their attributes were the frequencies of amino acids, the structural properties of proteins and a number of propensity scores. With all these attributes, the AUC was 0.55 and the accuracy was 70.6%. If only the best attributes were selected, the AUC and the accuracy increased to 0.59 and 80.4%, respectively. This large increase (in accuracy) may be due to the fact that apparently the whole data set was used in attribute selection instead of only the training set.

In this manuscript the analysis is based on a large data set of EAR determined by the high-density peptide array technology platform at the Rostock Epitope Screening Center (RESC) [14], [15], [24]. The peptide arrays were probed with commercially available intravenous immunoglobulin preparations (IVIG). IVIG is a purified IgG fraction usually prepared from the serum of between 1,000 and 15,000 healthy donors per batch and is intended for medical use in a number of conditions [25], [26]. see File S1. The data set was used to compare two computational methods for predicting the occurrence of continuous B cell epitopes. The methods were based on machine learning and the use of position weight matrix (PWM) analysis. Epitope classification was then related to predictions of MHC class I and class II binding and known X-ray structures of antigen-antibody complexes.

## Materials and Methods

### Peptide Array Data

Our epitope profiling methods are described in detail in [14] [15]. Different batches of high-density peptide microarrays (each carrying about 5,000 individual, usually 15mer, peptides in triplicate) were employed to screen for EAR. The chip content consisted of peptides mostly derived from human proteins supplemented with random peptides, peptides from non-human proteins or those with mutated sequences or non-standard amino acids (File S2). The peptide chips were incubated with commercial intravenous immunoglobulin fractions (IVIG). IVIG preparations from several commercial vendors were used (Omrix, Israel; Sclavo Vena NIV, Italy; Tegeline, Laboratoire Français du Fractionnement et des Biotechnologies LFB, France; Octagam, Octapharma GmbH, Langenfeld, Germany; Intratect, Biotest Pharma GmbH, Dreieich, Germany). More detailed informaton on the statistics of the used samples as well as on the nature of IVIG is given in File S1. For staining of the peptide chips, IVIG was usually used at 0.125 mg/ml. Bound antibodies from the IVIG preparations were visualized by processing the arrays with a fluorescently tagged secondary antibody, a goat Fab-fragment antibody directed against the Fc part of human IgG [14]. The data depth of scanned images was 16-bit, i.e. signals ranged from 0 to 65,535. Intensities given in the manuscript and in the supplements represent the average signals of the triplicates. If a peptide was measured in more than one experiment, the highest average signal intensity reached was further used. In each experiment, one chip was probed with secondary antibody alone to determine its background staining irrespectively from IVIG primary antibodies. Information on the analyzed peptide sets, the distribution of the signals and the reproducibility of the measurements are provided in File S2.

### Data Set Preparation

**Figure 1:**
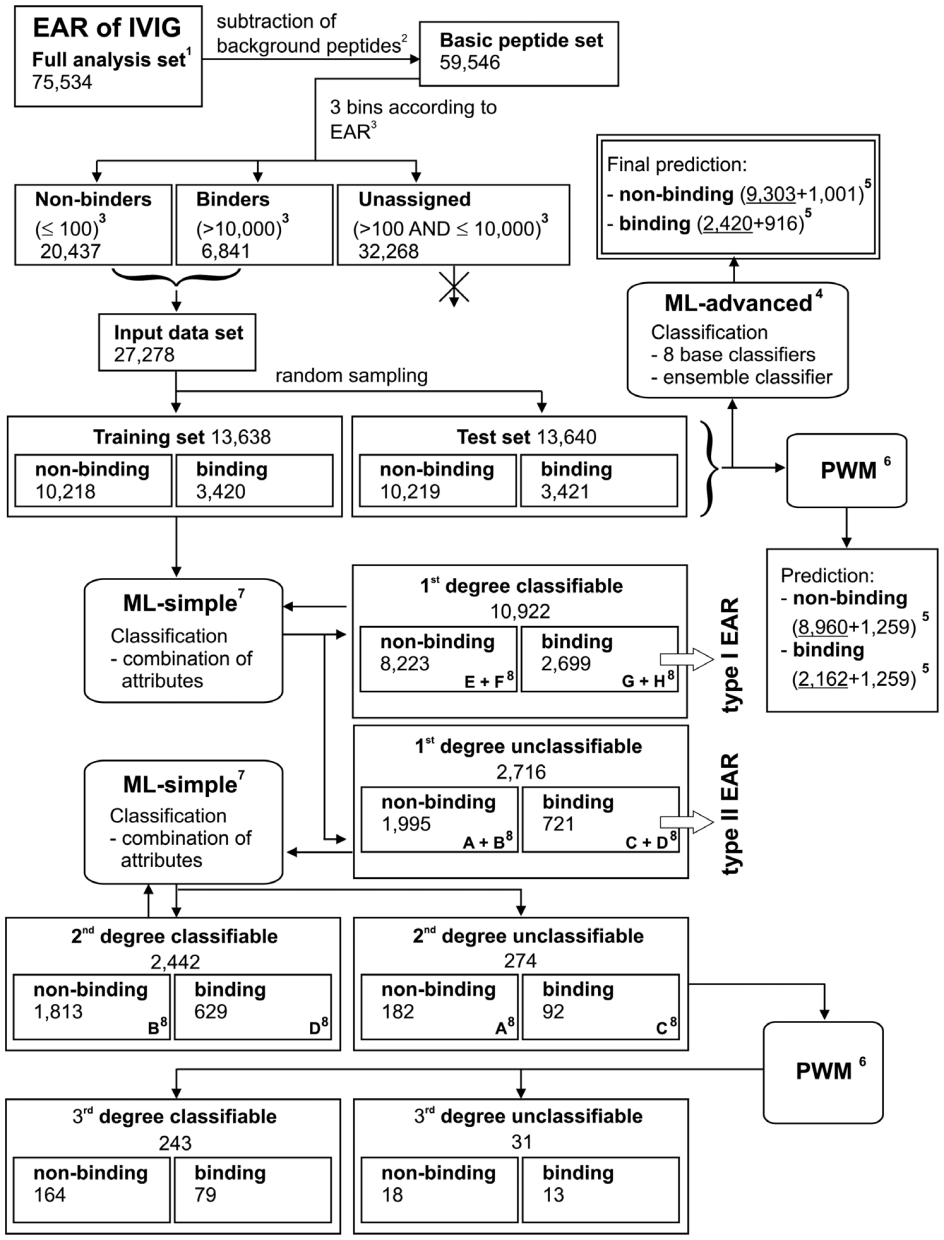
Data set preparation and computational workflow for the prediction of epitope-antibody-reactivities (EAR) determined for IVIG antibodies. Rectangles represent groups of peptides (numbers in each group are indicated), boxes with rounded corners indicate the applied classification approaches. ^1^All peptides printed on the microarrays ^2^Removal of false positive (binding) peptides (e.g. those reactive with secondary antibodies) ^3^Separation of peptide set according to signal intensities of EAR into non-binders, binders and unassigned peptides ^4^Classification approach ML-advanced = machine learning with an ensemble classifier ^5^Number of peptides predicted to be non-binding/binding, separated into those predicted correctly (underlined) and incorrectly ^6^Classification approach PWM = position weight matrix ^7^Classification approach ML-simple = simplified machine learning using human-understandable attributes ^8^Capital letters A–H indicate subsets of peptides assigned in supplementary information table S1 and explained there in the legend.

The data set preparation with the associated workflow is illustrated in Figure 1. In total, the EARs of 75,534 peptides (full analysis set) were measured. From those, all peptide sequences were removed whose signals from the secondary antibody alone were larger than signals from IVIG. These signals, designated non-specific background reactivities, are due to direct binding of secondary antibodies to specific peptide sequences. This operating procedure resulted in a basic peptide set of 59,546 sequences. These peptides were further divided into three categories with respect to signal intensities: 6,841 peptides with signals above 10,000 after subtraction of background values of secondary antibodies alone are designated “binders”; at the opposite end, 20,437 peptides with intensities below or equal to 100 after background subtraction are designated “non-binders”. These two groups are considered to be of high confidence with respect to recognition or non-recognition of antibodies present in IVIG preparations forming the input data set (see File S2 listing signal intensity distributions and the statistics of the peptide content). The group with in-between signals has not been analyzed further to exclude peptides that run the risk of being wrongly assigned. The peptides that bind antibodies are considered to contain at least one epitope, i.e., one antibody binding site. The input data set was split in half by random sampling to form a training and a test set (see File S2 for the procedure). The training set consisted of 3,420 peptides that were reactive with antibodies (“binding”) and 10,218 peptides that were not (“non-binding”). The test set consisted of 3,421 binding and 10,219 non-binding peptides, respectively. Most of the peptides studied were 15 amino acids in length (12,436 in the training, 12,420 in the test set). More information on sequence and origin of the peptides of the training set along with their measured signals are listed in Table S1. Their assignments to different classification groups defined in this paper are also indicated. The test set peptides are provided in a similar way in Table S2. This table includes prediction scores. The training and test sets contain roughly three times more non-binding than binding peptides. Such imbalanced data sets might reduce the performance of classifiers trained by machine learning algorithms [27]. To handle imbalanced data sets, two methods were applied, random oversampling and undersampling. The first method duplicates randomly chosen data in the smaller class (binding in our case). The second method removes randomly chosen data from the larger class (non-binding in our case). These two methods, while simple, were shown to outperform more sophisticated ones [27]. Both methods of over- and undersampling perfectly balanced both classes. When training the final classifier for epitope prediction, oversampling was used to take advantage of all information contained in the training set. When choosing the best attributes and machine learning algorithms, undersampling enabled fair comparison while offering greater speed and simplicity.

### Generation of Position Weight Matrices (PWMs)

The peptide sets were divided into binding and non-binding, and for each group a 20×15 PWM (20 standard amino acids×15 peptide positions) was constructed that described the frequency of each amino acid at each position [28]. Next, these primary PWMs were used to construct a ratio PWM containing the ratios of the frequencies of the amino acids at each position in the “binding” versus the “non-binding” PWM. A ratio higher than one corresponds to a more enriched amino acid at this position in “binding” peptides, while a value smaller than one means the opposite. A higher ratio thus also indicates that the amino acid is more likely to be present in epitopes. This ratio is termed “epitope propensity”. Position-dependent propensity values were calculated by averaging the ratio PWM values for each amino acid for all positions. Individual peptide sequences were ranked by computing PWM scores. Such scores are calculated by multiplying the ratios of the relative frequencies of each amino acid at each position in a peptide sequence.

### Workflow of Machine Learning with an Ensemble Classifier (ML-advanced)

An ensemble approach was used for epitope prediction. The training was done in two steps. In the first step, each peptide *p_i_* from the training set was first represented by eight attribute vectors *a*_1_(*p_i_*), …, *a*_8_(*p_i_*). Then eight base classifiers *C*_1_, …, *C*_8_ were trained, one for each attribute set. Each of the base classifiers used one of the attribute vectors as input and returned the probability that the peptide represented by the attribute vector contains an epitope. In the second step of machine learning, the probabilities returned by the base classifiers formed a new attribute vector: *a*_M_(*p_i_*) = [*C*_1_(*a*_1_(*p_i_*)), …, *C*_8_(*a*_8_(*p_i_*))]. To assign this new attribute vector to each peptide, five-fold cross-validation was used. The training set was split into five subsets, the base classifiers *C*_1_, …, *C*_8_ were trained on four of them and then used to estimate the epitope probabilities for the last one. This was repeated five times with a different split each time, so that all the peptides were assigned the probabilities. Finally, a meta-classifier *C*_M_ was trained on *a*_M_(*p_i_*) to compute the final probability that a peptide contains an epitope.

The testing of the classifier for epitope prediction followed the same two-step procedure as training. Each peptide *q_i_* from the test set was represented by eight attribute vectors *a*_1_(*q_i_*), …, *a*_8_(*q_i_*) and classified with the eight base classifiers *C*_1_, …, *C*_8_. The outputs of these classifiers were joined into a new attribute vector *a*_M_(*q*_i_) and classified with the meta-classifier *C*_M_, returning the final probability *C*_M_(*a*_M_(*q_i_*)) that the peptide *q_i_* contains an epitope. The procedure we describe is similar to stacking [29], [30]. The difference is that in stacking, all base classifiers are trained on the same attribute vectors, however, different machine learning algorithms are used. Stacking thus attempts to exploit the advantages of multiple machine learning algorithms, whereas our procedure in addition takes advantage of multiple ways to represent the data. This approach can, however, use multiple learning algorithms as well since a different algorithm can be employed to train each base classifier, including another level of stacking. The data set on which the classifiers were trained was balanced using random oversampling for all base classifiers. The meta-classifier was trained on both, the balanced and the original (imbalanced) training set.

Each of the eight attribute vectors used in machine learning consisted of different attributes:

- Frequencies of amino acids in the peptide (e.g., the frequency of alanine)
- Differences between the frequencies of the amino acids (e.g., the frequency of alanine minus the frequency of cysteine)
- Frequencies of the subsequences of the peptide up to a certain length (e.g., the frequency of alanine-alanine-cysteine)
- Physico-chemical properties of the amino acids (e.g., the average acidity)
- Frequencies of amino acid classes based on their physico-chemical properties (e.g., the frequency of acidic amino acids)
- Frequencies of the subsequences consisting of amino acid classes instead of individual amino acids (e.g., the frequency of acidic-neutral-acidic-neutral)
- Frequencies of pairs of amino acids with a certain distance between them (e.g., the frequency of the pair (alanine, cysteine) with the distance 3 between them)
- Frequencies of amino acids occurring at a certain distance from the first position in the peptide (e.g., the frequency of alanine at the distance 3 from the first position).

The attributes are described in more detail in File S3 (2. Attributes for machine learning). Several parameters in our machine learning procedure have been tuned. Firstly, each of the attribute vectors has been computed in various ways, which were controlled by attribute parameters. Secondly, each of the base classifiers has been trained by various machine learning algorithms, each of which has its own parameters – more on this in File S3 (4. Machine learning algorithms). And thirdly, the same is true for the meta-classifier. The tuning procedure is described in File S3 (3. Parameter tuning).

### Simplified Machine Learning Using Human Understandable Attributes (ML-simple)

The ensemble approach was designed for maximum prediction accuracy. However, for (qualitative) analysis and interpretation, a more simplified approach was applied. It uses only frequencies of amino acid subsequences up to length 5 (including the frequencies of single amino acids) and physico-chemical properties of amino acids as attributes because they are readily interpretable. To obtain a human-understandable classifier, the RIPPER machine learning algorithm was applied [31]. It produces rules with the following form:

IF (*a*_1_<*val*_1_) AND (*a*_2_≥*val*_2_) … THEN *class* = binding

…

ELSE IF (*a_n_*_–1_<*val_n_*_–1_) AND (*a_n_*≥*val_n_*) … THEN *class* = binding

ELSE *class* = non-binding

The letters *a_i_* indicate the attributes, *val_i_* the values these attributes may take and *n* the total number of attribute comparisons. Each rule applies to a certain number of peptides and each rule classifies a subset of them correctly. We found that even such a rule set is difficult to read, mostly because each attribute occurs in multiple rules, so we provide a summary instead. Each row of the summary consists of three items. The first is the attribute. The second is whether the attribute should be lower or higher than some value in order for the class to be binding (<*val_i_* or >*val_i_*, the exact value is omitted). The third item is the percentage of peptides that the rules containing this attribute classified correctly, out of all the binding peptides in the data set. This item signifies the importance of the attribute. With the intention to reach a balance between simplicity and accuracy, the human-understandable attributes were used in combination with logistic regression machine learning algorithms (File S3, 4. Machine learning algorithms).

### Presentation of Performance Measures

The epitope prediction performance is presented in terms of the classification accuracy and the area under curve (AUC) of receiver operating characteristic (ROC). The accuracy is defined as the number of correctly classified instances divided by the total number of instances. As in case of our data set, the score may be somewhat misleading since the numbers of instances belonging to each class are unequally distributed. The AUC is not sensitive to class imbalances and is a more general measure than the accuracy itself. The details of both measures are explained in File S3 (5. Epitope prediction performance measures).

### Prediction of MHC Peptide Binding

The NetMHC 3.2 [32] and NetMHCIIpan 1.1 server [33] online services were used to predict whether the peptides under study can be presented by MHC class I and class II complexes. These servers have been chosen because of their high web-based functionalities, availability, common use and high acceptance in the scientific community. The ten most common MHC alleles have been taken for analysis, see File S4 (3. MHC class I and class II prediction servers). Individual outputs of the servers for the peptides of the training set are given in Table S1. The MHC servers set a value of >0.5 as indication for binding. To compare predictions of both servers with measures of our peptide arrays, we plotted ROC curves for both MHC classes. The binding/non-binding classifications derived from peptide array analysis were taken as true values. This allowed us to express the degree to which the MHC predictions agreed with our peptide array measures.

### Analysis of PDB Structures

In total, 509 crystal structures of antibody and protein antigen complexes have been identified in Protein Data Bank and IMGT/3DStructure-DB (status January 2010). High quality protein complexes at resolutions of lower than 3.0 Å showing protein complexes with peptide lengths of more than 25 amino acids were selected, the similarity in binding of interfacial residues was mapped and redundant complex structures were removed. If more than 20% interfacial residues turned out to be identical within comparable antibody-protein complexes, the one with higher crystallization resolution was taken. The final data set of antibody/protein antigen complexes was composed of 81 crystal structures. Their PDB IDs are listed in Table S4. Amino acids of the antibody-bound peptide that made up an epitope were determined from the solvent accessible surface areas at the epitope-paratope interface as described previously [34]. The enrichment of amino acids in these epitopes was computed as follows: For each amino acid its cumulative count in an epitope was divided by the total number of residues in that epitope. Then these values were averaged over all epitopes and the percentage of each amino acid’s frequency was determined. For combined frequencies, the relative frequencies of single amino acids in epitopes were averaged.

## Results

Prediction of peptide binding to antibodies present in IVIG was based on a data set containing EAR for 75,534 distinct continuous peptide sequences. From this full analysis set, 9.05% (6,841 of 75,534 peptides) showed high confidence EAR with background-corrected signals of greater than 10,000. They were derived from altogether 870 different human protein sequences. Though these IVIG antibodies have been collected from thousands of sera of healthy donors, these data show that a large number of peptides of human origin are reproducibly recognized by IVIG samples. With the help of this data set, machine learning and PWM approaches have been applied to determine how well EAR can be predicted. A scheme of the workflow that provides a directed walkthrough of each step conducted is outlined in Figure 1. First an elaborate machine learning approach was established which used an accurate but difficult to interpret ensemble classifier (ML-advanced). Then, a simplified machine learning approach was conducted using human-understandable attributes and rules (ML-simple). The principle advantage of the additional PWM approach was that it is more readily readable and facilitates to point to position effects and amino acid patterns that can be experimentally explored.

### Epitope Prediction

**Figure 2:**
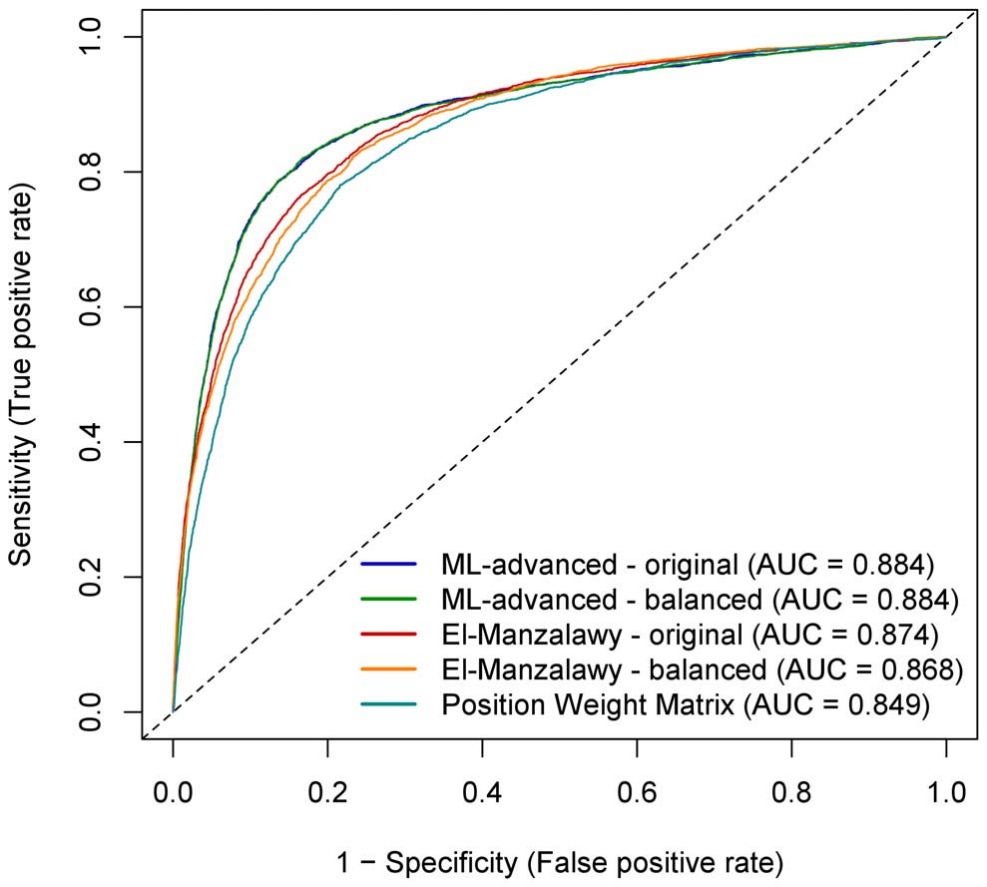
Performance comparison of the tested classifiers for IVIG binding prediction on the test set peptides. ROC analysis for our machine learning approach with an ensemble classifier (ML-advanced), the machine learning method of El-Manzalawy et al. (2008) [22], [23] and a PWM approach using an PWM derived from the training set. Both machine learning approaches were trained on the original training set (“original”: three times more non-binding than binding peptides) and on the balanced training set (“balanced”: equal number of binding and non-binding peptides) and finally applied on the test set. AUC values are indicated as well. Note that the curves based on the original and balanced training set of our ML-advanced method show almost complete overlap.

Classifiers for epitope prediction were trained on the training set and applied to the test set. Both sets contained three times more non-binding than binding peptides, but this may not necessarily be the case for potential new data. Thus, we investigated the performance of classifiers trained on the original training set as well as on balanced data sets containing an equal number of binding and non-binding peptides. In the first scenario, the proportion of binding vs. non-binding peptides in the test set is considered to be the same as in the training set. In the second scenario, binding and non-binding peptides are assumed to be equally likely in training and test set. Firstly, the performance of the eight base classifiers used in machine learning was evaluated. Table 1 shows the performance achievable with relatively simple classifiers providing a baseline for the final ensemble classifier. As shown by the AUC and the accuracy of each of the base classifiers, their performances were quite similar. Thus, none of them could be excluded from the ensemble. The final ensemble classifier ML-advanced was compared to the PWM approach using the unbalanced data set as well as to the classifier by El-Manzalawy et al. [22], [23] that was taken as state-of-the-art in epitope prediction. AUC scores and accuracies characterized ML-advanced as slightly better compared to the machine learning according to El-Manzalawy when trained on both, the original and balanced training sets (Table 2). Details in terms of the number of correctly predicted peptides for all three approaches are shown in Table 3. The corresponding ROC curves are visualized in Figure 2, together with those derived from PWM classification of the training set. They corroborated the slightly better performance of our ML-advanced approach compared to the El-Manzalawy machine learning and illustrated the superiority of machine learning in comparison to PWM analysis. Predictions for individual peptides in the training and test set can be found in Table S1 and Table S2, respectively. The ensemble classifier of ML-advanced outperformed the base classifiers (compare results in Table 2 with the balanced results in Table 1). The difference in terms of the AUC is modest, ranging from 0.01 to 0.02, but the difference in terms of the accuracy is larger, at least by 2.5 percentage points. To determine the attributes that enable the prediction of “binding”, peptides of the training set were classified in a machine learning approach named ML-simple (see Methods) by using attributes reflecting human-readable rules. The rule summary (Table 4) shows that attributes significantly associated with prediction of “binding” were in particular high aromaticity, low polarity and high tyrosine content. However, the moderate percentages of correctly classified peptides for the individual attributes illustrate once more the advantage of using an ensemble approach for the most accurate prediction.

**Table 1:** AUC and accuracy of binding prediction for IVIG antibodies using the eight base classifiers that are a part of the ML-advanced machine learning approach*.

Attribute vector	AUC	Accuracy
Frequencies of amino acids	0.870	80.7%
Difference between frequencies	0.868	80.3%
Frequencies of subsequences	0.867	80.5%
Physico-chemical properties	0.873	81.2%
Frequencies of amino acid classes	0.866	80.5%
Frequencies of subsequencesof classes	0.865	80.6%
Frequencies of pairs of amino acids	0.873	81.2%
Frequencies of amino acids at adistance from first position	0.863	80.3%

* The base classifiers were cross-validated on the balanced training set (equal number of binding and non-binding peptides). Balanced data was chosen because the base classifiers were always trained on balanced data, the original data were only used in the final step of merging their results. The training set was chosen instead of the test set because comparing various methods on the test set can lead to selecting them based on those results, which defeats the purpose of an independent test set.

**Table 2:** AUC and accuracy of epitope predictions on the test set*.

	Original training set		Balanced training set	
	ML-advanced	El-Manzalawy	ML-advanced	El-Manzalawy
**AUC**	0.884	0.874	0.883	0.868
**Accuracy**	85.9%	83.9%	83.7%	82.8%

* Comparison of our final ensemble classifier (machine learning approach ML-advanced, see Figure 1) to the classifier by El-Manzalawy et al. [22] [23]. Both were trained on the original training set (three times more non-binding than binding peptides) and on the balanced training set (equal number of binding and non-binding peptides) and finally applied on the test set. The results on the balanced training set can directly be compared to those in Table 1.

**Table 3:** Performance of the classifiers illustrated by confusion matrices of the prediction of IVIG binding on the test set.

		Classifiers trained on:			
		Original training set^a^		Balanced training set^a^	
Classifier^b^	Prediction	Actual binders^c^	Actual non-binders^c^	Actual binders^c^	Actual non-binders^c^
ML-advanced	Binding	2,420	916	2,735	1,539
	Non-binding	1,001	9,303	686	8,680
2-PWM	Binding	2,162	1,260	n.d.^d^	n.d.^d^
	Non-binding	1,260	8,958	n.d.^d^	n.d.^d^
El-Manzalawy	Binding	2,253	1,033	2,416	1,453
	Non-binding	1,168	9,186	1,005	8,766

a Training sets consist of either three times more “non-binding” than “binding” peptides (original) or an equal number of both groups (balanced).

b Our classifiers (ML-advanced, PWM with treshold 2.45; see Figure 1 for workflow) and the classifier of El-Manzalawy et al. [22] [23].

c Correct predictions are underlined.

d not determined.

**Table 4:** Rule summary for the whole training set when applying the simplified machine learning approach consisting of human-understandable attributes (ML-simple) for prediction of IVIG binding.

Attribute^a^	Low\high^b^	Classified correctly^c^
Aromaticity	High	53.8%
Polarity	Low	27.7%
Frequency of tyrosine	High	26.2%
Hydrophobicity	Low	22.5%
Frequency of arginine	High	19.7%
Summary factor 2	High	16.7%
Acidity	Low	11.4%
Preference for β-sheets	Low	4.3%
Summary factor 5	High	3.0%

a Details on the attributes, including the two summary factors “Summary Factor 2” and “Summary Factor 5” that combine 494 amino acid properties, are given in File S3 (2. Attributes for Machine Learning).

b Whether the rules state the value of the attribute should be high or low for a peptide to be binding.

c The percentage of binding peptides that were correctly classified by rules containing the attribute. This percentage roughly corresponds to the importance of the attribute.

### Stratification of Classifiable and Unclassifiable Peptides

While experimenting with various attributes and machine learning algorithms, we discovered that many of them can predict epitopes with an accuracy of around 80%. Not all classifiers misclassified the same peptides, which is why combining the classifiers into an ensemble improved the performance (as seen in Table 1 and Table 2). However, it seemed that 15–20% of the peptides resisted correct first round classification irrespective of the method used. To investigate this phenomenon further, we divided the peptides of the training set into “1^st^ degree classifiable” and “1^st^ degree unclassifiable” and analyzed each group separately. For this, we used the interpretable attributes, namely the frequencies of amino acid subsequences up to length 5 and physico-chemical properties of amino acids, and logistic regression as the machine learning algorithm (note, that these attributes and this algorithm were used in all the experiments in this subsection). The division of the data set was carried out by splitting the whole original training set into five subsets. Each of the subsets was divided into classifiable and unclassifiable peptides by a classifier trained on the remaining four subsets. Those that were classified correctly were considered classifiable and the rest unclassifiable. All five classifiable subsets were then merged into a single set of 1^st^ degree classifiable peptides and the same was done for the 1^st^ degree unclassifiable ones. The assignment of particular peptides to these classes is listed in Table S1. Finally, a classifier was cross-validated on the 1^st^ degree classifiable and unclassifiable peptides separately. The respective performances of these classifiers are shown in Table 5. The accuracy on all peptides of the training set reached 83%, which may be expected based on Table 1 and Table 2. The accuracy on the 1^st^ degree classifiable peptides was close to 100%, which was also as expected. The value did not reach 100% because the classifier was exclusively trained on the 1^st^ degree classifiable peptides, whereas the classifier that divided the peptides into classifiable and unclassifiable was trained on all peptides of the training set. However, the accuracy on the 1^st^ degree unclassifiable peptides was also high (91.5%), which was not expected. To understand this phenomenon, we introduced rules for 1^st^ degree classifiable and unclassifiable peptides separately. The rule summary is provided in Table 6, and the complete rule set in File S3 (6. EAR Rules). Table 6 explains the unexpectedly high classification cross-validation accuracy on the unclassifiable peptides: The 1^st^ degree classifiable and unclassifiable peptides are exactly the opposite of each other with respect to the attributes most useful for classification. For example, if a peptide is classifiable, it is likely to bind if it has a high aromaticity; if it is unclassifiable, it is likely to bind if it has a low aromaticity. Because of this opposite behavior, a classifier that correctly classifies one of the two groups must fail on the other. The classifier that was trained on the whole training set correctly classified the larger group (classifiable) and failed on the smaller one (unclassifiable). However, when the classifier that was trained on the 1^st^ degree unclassifiable peptides was used, it no longer faced the contradiction between the groups and thus performed well.

**Table 5:** Performance for the whole training set, 1^st^ degree classifiable and 1^st^ degree unclassifiable peptides employing the simplified machine learning approach with human-understandable attributes (ML-simple) for prediction of IVIG binding*.

	Training set	1^st^ degree classifiable	1^st^ degree unclassifiable
**AUC**	0.860	0.999	0.956
**Accuracy**	83.0%	98.8%	91.5%
**Number of peptides**	13,638	10,922	2,716

* Comparison of AUC and accuracy when the classifier was 10-fold cross-validated on all the peptides in the original training set, or on peptides that the first classifier classified correctly (1^st^ degree classifiable) or incorrectly (1^st^ degree unclassifiable), respectively. All classifiers used the interpretable attributes and logistic regression.

**Table 6:** Rule summary for the 1^st^ degree classifiable and unclassifiable peptides employing the simplified machine learning approach with understandable attributes (ML-simple) for the prediction of IVIG binding.

Attribute^a^	1^st^ degree classifiable		1^st^ degree unclassifiable	
	Low\high^b^	Classified correctly^c^	Low\high^b^	Classified correctly^c^
Aromaticity	High	74.3%	Low	53.3%
Polarity	Low	58.7%	High	27.5%
Frequency of arginine	High	31.5%	Low	34.0%
Frequency of tyrosine	High	20.7%	Low	16.9%
Summary factor 5	High	15.1%	Low	15.2%
Antigenicity	High	7.3%	Low	8.7%
Hydrophobicity	Low	4.7%	High	6.5%
Frequency of histidine	Low	3.9%		
Frequency of cysteine			Low	10.4%
Preference for reverse turns			High	10.4%
Occurrence in turns			Low	10.4%
Frequency of alanine			High	8.7%

a Details on the attributes are given in File S3 (2. Attributes for Machine Learning).

b Whether the rules state the value of the attribute should be high or low for a peptide to be binding.

c The percentage of binding peptides that were correctly classified by rules containing the attribute. This percentage roughly corresponds to the importance of the attribute.

### Propensities of Amino Acids

**Figure 3:**
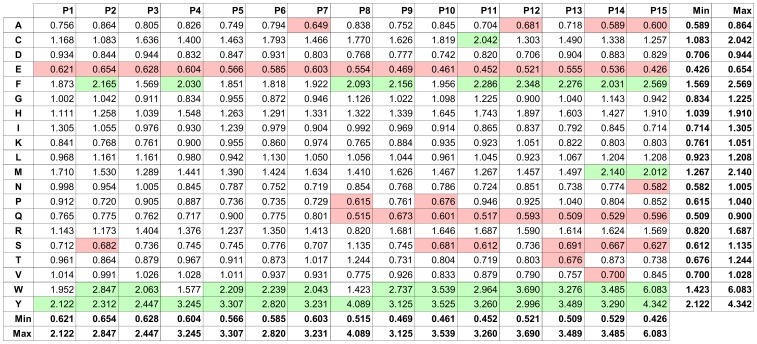
Ratio PWM of amino acids in peptides that were “binding” versus those that were “non-binding” to IVIG antibodies. The PWM has been created using all 15-mer peptides from the training set. Each component of the PWM corresponds to the ratio of the frequency of the occurrence of a given amino acid (row) at a given peptide position (column) in “binding” vs. “non-binding” peptides. Amino acids Y, W, and Y have the highest binding ratios, whereas amino acids E, Q, and N have the lowest ratios. Green shading represent amino acids that are more abundant in “binding” peptides than in “non-binding” ones (threshold>2). In contrast, red shading labels amino acids that are more abundant in “non-binding” peptides (threshold <0.7). The range of values in each column and row is indicated by minima (Min) and maxima (Max).

**Figure 4:**
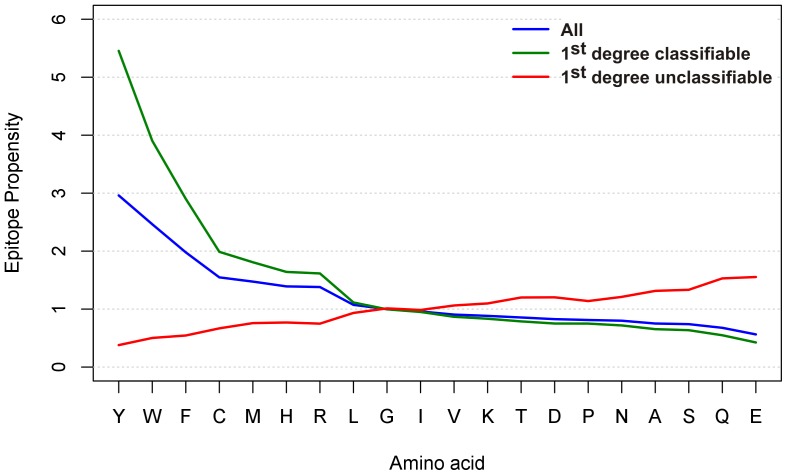
Analysis of amino acid enrichment in IVIG “binding” versus “non-binding” peptides for all, the 1^st^ degree classifiable and 1^st^ degree unclassifiable peptides of the training set. Amino acid propensities for the indicated groups were determined by dividing the frequency of amino acids in “binding” peptides (recognized by IVIG) by the frequency of amino acids in non-binding peptides. Results are sorted by decreasing epitope propensity assigned to the whole training set group. A propensity score >1 means that an amino acid is more likely to occur in the “binding” peptides, a score <1 more frequent in the “non-binding” ones, respectively. The analysis is position-independent.

Although inferior in prediction efficacy, the ratio PWM of the amino acid distribution in binding versus the non-binding peptides of the training set helped to pinpoint amino acids important for IVIG binding. The matrix indicates a higher frequency of tyrosine (Y), tryptophan (W) and phenylalanine (F) in binding and a higher incidence of glutamic acid (E) and glutamine (Q) in non-binding peptides (Figure 3). Clear-cut position-specific effects for these amino acids within the peptides were not observed. However, the detected ranges in values (indicated by minimum and maximum) in the PWM for many amino acids raise the possibility that position effects may exist: it is conceivable that even strong EARs of particular antibodies with a particular binding pattern have been obscured due to overlapping binding of thousands of different antibodies. To investigate the properties of 1^st^ degree classifiable and unclassifiable peptides in more detail, we investigated the epitope propensities (ratios of the frequency in binding vs. non-binding peptides) of the 20 standard amino acids in each group separately. 1^st^ degree classifiable and unclassifiable peptides show an opposite behavior in terms of epitope propensities (Figure 4): those amino acids that have higher epitope propensities in 1^st^ degree classifiable peptides (and to a lesser degree in all peptides), have lower propensities in unclassifiable peptides, and vice versa. Numeric values of epitope propensities are listed in Table S3. The analysis demonstrated a strong overrepresentation of amino acids Y, W and F in the “binding” subset of 1^st^ degree classifiable peptides. In contrast, in particular Q and E as well as serine (S), alanine (A) and asparagine (N) were more frequent in the “non-binding” peptides. Both observations were already visible in the graph for the whole training set and coincided with the PWM on the whole training set described above (Figure 3). PWM analysis on the 1^st^ degree classifiable \ unclassifiable peptides corroborated these rules and epitope propensities as well (File S4, 2. Additional PWM Results).

### Application of PWM Analysis to Restricted Peptide Sets

**Figure 5:**
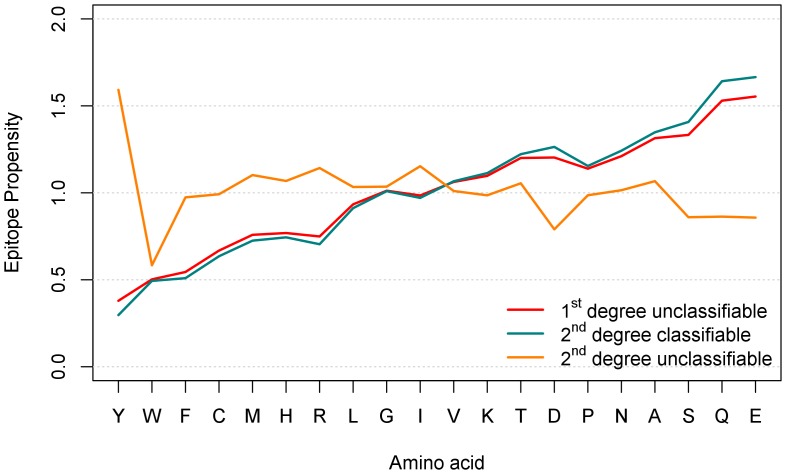
Analysis of amino acid enrichment in IVIG “binding” versus “non-binding” peptides for the 1^st^ degree unclassifiable peptides of the training set after a further split into 2^nd^ degree classifiable and unclassifiable ones. For legend, see Figure 4. The red curve is the same as in Figure 4, however, the scale is different here.

**Figure 6:**
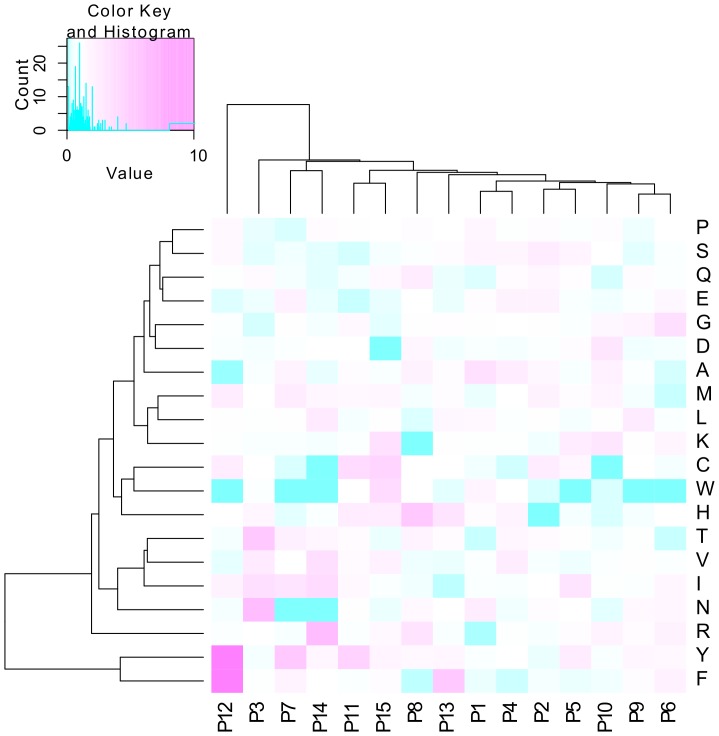
Ratio heat map based on amino acid propensities of IVIG “binding” versus “non-binding” peptides for the 2^nd^ degree unclassifiable peptides. The rows represent the individual amino acids, the columns the positions within the 15mer peptide. The heat map color reflects the ratio between the PWM values (frequency of the occurrence of a given amino acid at a given peptide position) for the “binding” and the “non-binding” peptides in the set. Pink color indicates high propensity (overrepresentation in “binding” peptides), while blue color indicates low propensity (underrepresentation in “binding” peptides). Standard hierarchical clustering using Euclidean distance was performed on rows and columns.

Since 8.5% of the 1^st^ degree unclassifiable peptides were not classified correctly when a classifier was cross-validated on them, we further divided the peptides into 2^nd^ degree classifiable and unclassifiable (Table 7). Again, a classifier was cross-validated on each of the 2^nd^ degree subsets separately. The accuracy on the 1^st^ degree unclassifiable\2^nd^ degree classifiable peptides was 97.5% as expected, while the accuracy on the 1^st^ degree unclassifiable\2^nd^ degree unclassifiable peptides was only 65%. This is as good as simply classifying all of them as non-binding by default (66.4%). Propensity graphs showed that the amino acid distributions were almost indistinguishable for all 1^st^ degree unclassifiable and 1^st^ degree unclassifiable\2^nd^ degree classifiable peptides (Figure 5), likely because both sets largely overlap (roughly 90%). However, the amino acid distribution of the 274 residual 1^st^ degree unclassifiable\2^nd^ degree unclassifiable peptides was unlike any of the groups investigated so far. In contrast to the failure of the machine learning approach on these 274 peptides, the PWM-derived heat map (Figure 6) outlines specific amino acids at distinct peptide positions that distinguish “binding” from “non-binding” peptides. This PWM classification on a limited number of peptides left only very few peptides unclassifiable (see “3^rd^ degree classifiable” versus “3^rd^ degree unclassifiable” peptide sets in Figure 1). The high AUC of 0.962 and accuracy of 88.7% (ROC curve provided in File S4 (2. Additional PWM results)) indicate that amino acid specific position effects specified in small EAR data sets play a major role in epitope recognition and antibody binding.

**Table 7:** Performance for 1^st^ degree unclassifiable peptides, further split into 2^nd^ degree classifiable and unclassifiable peptides employing the simplified machine learning approach with understandable attributes (ML-simple) for prediction of IVIG binding*.

	All 1^st^ degreeunclassifiable	1^st^ degree unclassifiable\2^nd^ degree classifiable	1^st^ degree unclassifiable\2^nd^ degree unclassifiable
**AUC**	0.956	0.992	0.683
**Accuracy**	91.5%	97.8%	65.0%
**Number of peptides**	2,716	2,442	274

* Comparison of AUC and accuracy when the classifier was 10-fold cross-validated on peptides of the 1^st^ degree unclassifiable set or on its peptide subsets that the first one classified correctly (2^nd^ degree classifiable) or incorrectly (2^nd^ degree unclassifiable), respectively. All classifiers used the interpretable attributes and logistic regression.

### Evidence for Two Types of EAR

**Figure 7:**
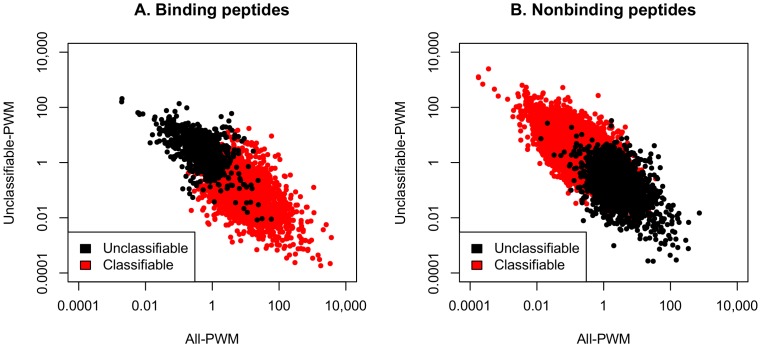
Distribution of peptides initially scored as 1^st^ degree classifiable and unclassifiable by ML-simple using PWM measures. Peptides of the training set were assigned to the groups 1^st^ degree classifiable and unclassifiable by our “simplified machine learning using human-understandable attributes” (ML-simple) approach. They were further divided into peptides reacting with IVIG (“binding”; panel A) or not reactive with IVIG (“non-binding”; panel B). In a next step each peptide was assigned values using two ratio PWMs. The x-axis values derive from a PWM that was based on all peptides present in the training set. They are calculated by multiplying the ratios of the relative frequencies of each amino acid at each position in a peptide sequence for the group “binding” (panel A) and “non-binding” (panel B), respectively. The y-axis values were calculated in the same way, however, only the 1^st^ degree unclassifiable peptides present in the training set were used as input of the PWM. Each peptide is represented by one dot. Peptides in red in panel A correspond to the type I EAR while those in black depict the type II EAR.

Our machine learning approaches initially identified two main classes of peptides, 1^st^ degree classifiable and unclassifiable peptides (see above; Table 5). In order to directly relate this classification to information on the amino acid composition, the characteristics of respective peptides were visualized with the help of two ratio PWMs in separate scatter graphs for the “binding” and “non-binding” peptides (Figure 7). The visualization indicated that the 1^st^ degree classifiable and unclassifiable peptides disperse into two distinguished groups for both, the “binding” and “non-binding” peptides, due to their opposite physico-chemical characteristics. Based on this behavior, we propose that there are two types of EAR for “binding” peptides: The designation Type I EAR describes the 1^st^ degree classifiable while Type II EAR indicates the 1^st^ degree unclassifiable peptides bound by IVIG antibodies. The amino acid propensity and PWM analyses argue that Type I EAR peptides are characterized by high Y, W and F and low E, Q, S, A and N amino acid content while for Type II EAR it is the other way around.

### PDB Epitope-paratope Structures

For validation of the existence of these two types of EAR, we looked at structural data in the Protein Data Bank PDB database describing epitope-paratope interactions. We calculated the combined relative frequency of the amino acids thought to be typical for Type I and Type II EAR within the epitopes of 81 complexes (see Table S4). For Type I EAR the analysis was focused on the presence of Y, W and F. For Type II EAR amino acids E, Q and N were taken. Amino acids S and A were not regarded since they appear to be generally enriched in protein-protein contacts [35] [36] and were expected to unspecifically increase the combined frequencies for Type II EAR. The structures 1TZI and 2DD8 have been identified to be particularly supportive for epitopes of Type I EAR: Both complexes display a high ratio of Y+W+F compared to N+Q+E in the residue compositions of both epitopes. Interestingly, both harbor Y and F (11.11% of Y and F for 1TZI, and 16.67% of Y and 4.17% of F for 2DD8) rather than W (0% for both structures). On the opposite side, N+Q+E content is zero for 1TZI and negligible for 2DD8 (0% of N and E, and 4.17% of Q). The top candidates for Type II EAR based on a strong overrepresentation of N+Q+E versus Y+W+F are represented in complexes 2FD6 and 2J4W. There are no amino acids Y, W and F in the epitopes of either of them. The average content of N+Q+E is 11.8% (17.65% of N, 11.76% of Q and 5.88% of E) in 2FD6 and 11.1% in 2J4W (6.67% of N, 0% of Q and 26.67% of E). All these examples illustrate that crystal structures of epitopes bound by antibodies can be identified that resemble peptides designated Type I and Type II EAR.

### Peptide Binding to MHC Class I and Class II Complexes

**Figure 8:**
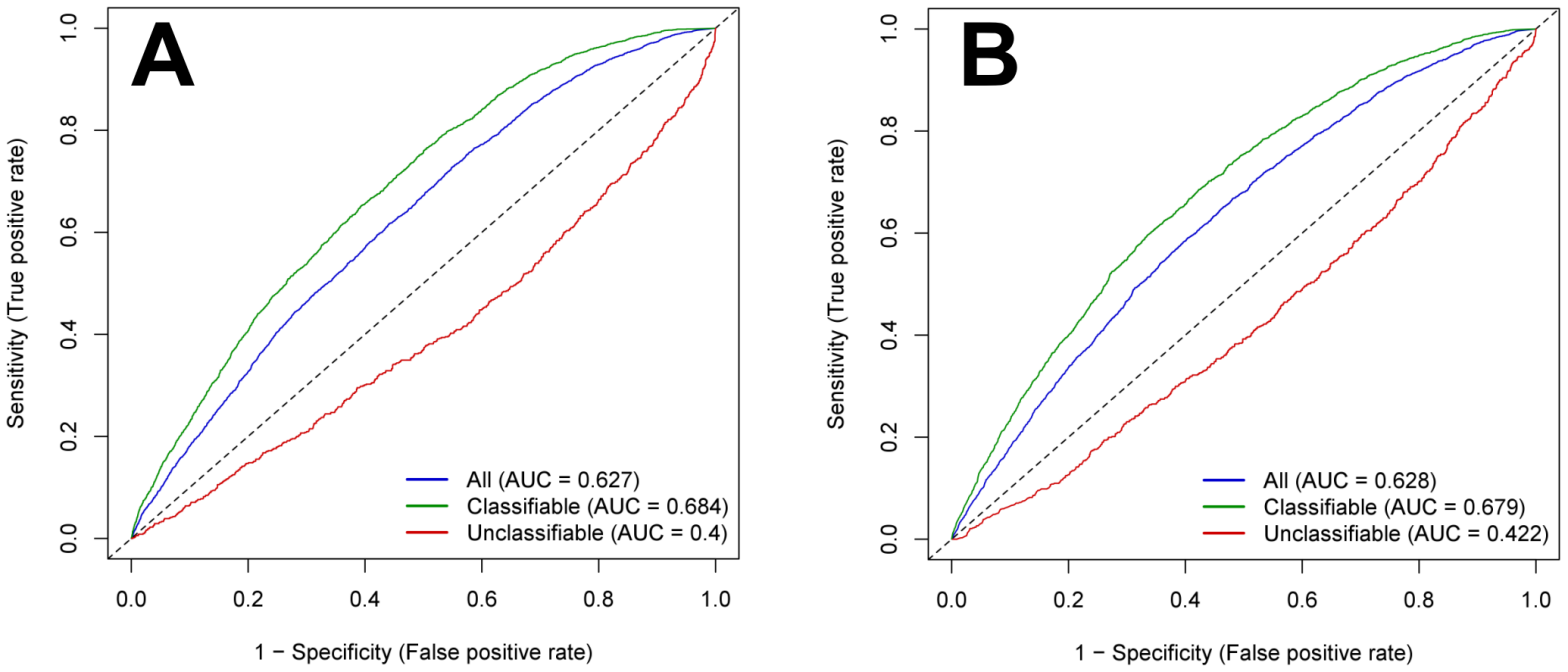
Performance chart for the assignment of peptides to the class “binding” to IVIG antibodies based on MHC binding. Separate ROC curves and AUC values for all peptides of the training set, the 1^st^ degree classifiable and unclassifiable peptides are presented. True positive peptides are those that are scored as MHC binding and at the same time were found to bind IVIG in the experiments. Predictions for MHC class II binding based on the NetMHCIIpan server (A) or for MHC class I binding based on the NetMHC server (B).

The NetMHC [32] and NetMHCIIpan [33] servers were used to predict the binding of the peptides in the training set to MHC complexes. The rationale of this analysis was that B cells do not commonly undergo a class switch from IgM- to IgG-producing B cells without T cell help. Determinants of peptides recognized by IVIG antibodies might have either been directly presented by MHC class II molecules to T cell receptors of CD4-positive helper T cells or indirectly by peptides derived from the same antigen/protein being engulfed by corresponding B cell receptor antigen complexes. The predicted peptide-MHC interactions were determined for all the peptides, and for the 1^st^ degree classifiable and unclassifiable peptides separately (Figure 8). The performance measures for MHC class II reached an AUC of 0.617, suggesting that peptides bound by antibodies can indeed be presented by MHC class II molecules. If performance was computed for the 1^st^ degree classifiable and unclassifiable peptides separately, the results were AUCs of 0.678 and 0.400, respectively. These results indicate that for the 1^st^ degree classifiable peptides, the prediction of MHC class II binding is even better than for the whole training set, while for 1^st^ degree unclassifiable peptides the performance dropped. Interestingly, for MHC class I peptide binding, the performance measures were very close to those for prediction of MHC class II binding (Figure 8). Possibly, Type I EAR binding rules are partly shared in peptide binding to MHC class I and class II complexes.

## Discussion

The human immune system is capable of generating around 10^10^ to 10^12^ different antibodies by genomic recombination. A recent estimation even reached an antibody repertoire of more than 10^16^ different immunoglobulins [5]. Due to the nature of how antibody repertoires are generated on the genome level, numerous of these antibodies most likely utilize mutual principles of epitope recognition – the reason why common EAR rules can be extracted and employed. In this paper, EAR predictions have been applied to stratify potential epitope sets that define two different types of IVIG-specific EAR. A set of IVIG-specific epitopes has been determined as starting point for future experimental and computational validation.

### Advanced Machine Learning for Epitope Prediction

The classifier trained by our ensemble machine learning approach (ML-advanced) for epitope prediction succeeded in most cases in surpassing the one that was selected as a benchmark (El-Manzalawy et al. [22] [23]). The difference was modest, but not insignificant considering that even relatively simple approaches perform quite well on our peptide data set. The better performance of our ML-advanced approach was documented in the number of correctly recognized binding as well as non-binding peptides both, when trained on the original and on balanced training sets (Table 3). When the classifiers were trained on the original training set, the number of mistakes on the binding peptides is larger, which may indicate that capturing the properties of binding peptides is more difficult than those of the non-binding peptides. Once the classifiers were trained on the balanced training set, both favored the identification of binding peptides because they are more numerous in the balanced training set than in the test set. However, the classifier used by El-Manzalawy et al. (2008) [22], [23] slightly outperformed our machine learning approach at high true and false positive (binding) rates (see ROC curves in Figure 2), which means when the number of recognized binding peptides will be maximized at the expense of misclassifying non-binding ones. The most interesting aspect of our ensemble classifier was probably in the way how the attributes were combined. Eight different attribute vectors were used to describe peptide characteristics, each of them consisted of a number of related attributes. Each vector gave fairly accurate epitope predictions, but some of their attributes failed on different peptide subsets, demonstrating that using only one attribute vector does not lead to an optimal solution. The alternative solution of using a single attribute vector had three disadvantages: (1) we could not have used the best machine learning algorithm for each attribute vector, (2) the combined vector could turn out to be too long – a problem for some machine learning algorithms, and (3) the attributes could not be combined in a “smart” way, which we could accomplish with the ensemble approach.

### Limits of Epitope Prediction

The design of a high-performance classifier for epitope prediction raises the principle question on the limits of such machine learning approaches. There may be representations of peptides, which could be used as attributes for machine learning, that capture all relevant epitope features for antibody binding and ignore all properties irrelevant for antibody binding. It appears that such a representation needs to include the information on whether a peptide belongs to the classifiable or unclassifiable group (at least 1^st^ degree, if not also 2^nd^). However, we do not yet know how to obtain this information without knowing the class of the peptide (binding or non-binding). It is also possible that one simply cannot do much better on a data set such as ours. Obviously, some peptides belong to groups that have common characteristics, which can be learned by machine learning algorithms, so they are classified correctly – these are the 1^st^ degree classifiable ones. The remaining peptides are not classified correctly because their broad characteristics point to the wrong classification, while the immune system classifies them based on more specific characteristics most likely by using different modes of antibody binding.

### Epitope Antibody Reactivities

Since antibodies are thought to recognize epitopes of around 8 to 12 amino acids in length [37], a 15-mer peptide in principle enables binding of more than one antibody, e.g. in different orientations to the same peptide molecule. Thus, each peptide in fact represents numerous epitope binding sites. Further, since assay signals for each peptide represent the sum of EAR to thousands of individual peptide molecules of the same sequence, different antibody species with similar binding specificities can contribute. When large EAR data sets are studied, it seems plausible that positional information remains obscured by antibodies with overlapping binding specificities that might have different properties. For instance, a peptide with one tyrosine at a certain position might attract antibodies that just recognize tyrosine residues irrespective of other surrounding amino acids, i.e. this EAR would be position-independent. Alternatively, such a peptide might bind to antibodies in a tyrosine position-dependent manner that appreciate the nature of specific amino acids in the neighborhood of this residue. Hence, bound classifiable and perhaps also bound unclassifiable peptides interact either with a group of distinct general-purpose antibodies each sharing common structural features or with a group of highly specific antibodies. In the latter case, position-specific epitope binding preferences might be obscured by the number of overlapping antibody binding specificities. Tailored peptide sets, e.g. with specific permutations at certain positions, well characterized antibody sets and streamlined computational approaches that focus on position effects might be applied in the near future to distinguish these kinds of antibody reactivities.

In the case that small numbers of peptides are studied, preferences of specific amino acids at specific peptide positions can be visualized by PWM analysis (see Figure 6). PWM approaches using small data sets reveal information on position effects as long as antibodies are forced to bind to one epitope position by restricting the size of linear peptides so that any type of sliding is prohibited. Thus, the remaining few 2^nd^ degree unclassifiable peptides in our data set might be recognized by quite structurally distinct antibodies limited in diversity, each one displaying diverse binding modes in an amino acid position-specific manner. In future studies, these assumptions can be tested as recently demonstrated [38].

### Type I EAR

The typical classifiable epitopes bound by IVIG antibodies (Type I EAR) share several properties expected from the literature: high frequency of tyrosine [16], low hydrophobicity [39] and high antigenicity (compare to Table 6). They have secondary structure features implemented in “high summary factor 2” implying the occurrence of turns, coils and bends [40], and low preference for β-sheets. Amino acids enriched in turns are common in epitopes [41]. The strongest trait of our Type I EAR peptides is their aromaticity (tyrosine, tryptophan and phenylalanine), which has been observed before to be relevant for epitopes [16]. Somewhat surprisingly, our bound peptides tend to be non-polar, while epitopes were described to be polar [16]. However, this is probably due to the emphasis on aromaticity, since three of the four aromatic amino acids are non-polar. Antibody generation giving rise to Type I EAR might directly be induced by T cell activation elicited by peptide binding to MHC class II complexes, see ROC curve of 1^st^ degree classifiable peptides that are predicted to be binding to MHC class II with a higher AUC score than all peptides of the training set (Figure 8).

### Type II EAR

A second class of epitopes are represented by 1^st^ degree unclassifiable peptides bound by IVIG (Type II EAR). Their properties are opposed to those of Type I EAR peptides, i.e. they are specifically enriched in polar amino acids asparagine, glutamine and glutamic acid and display low aromaticity. The polarity in this group here would fit previous epitope descriptions [16]. Antibodies binding to these peptides are suspected to have been generated independently of specific MHC-peptide presentation to T cell receptors, see ROC curve of 1^st^ degree unclassifiable peptides (Figure 8).

### Peptide Binding to MHC Class I and Class II Complexes

Since only short stretches of amino acids are presented by MHC molecules, MHC class I and class II peptide epitopes are more likely to resemble continuous epitopes in respect to MHC binding and T cell receptor recognition. The MHC epitope prediction performed on peptides classified by machine learning indicated that the binding of each set of peptides to MHC class I and class II might share common principles of protein-protein interaction modes with antigen-antibody-recognition. With respect to the two MHC classes I and II, both types of peptides (1^st^ degree classifiable and unclassifiable) show a similar behavior. However, while 1^st^ degree classifiable peptides show higher AUC scores for binding prediction to MHC class I and II than the peptides of the whole training set, the 1^st^ degree unclassifiable peptides perform worse. An AUC score of smaller than 0.5 in MHC peptide binding prediction indicates that peptides bound by Type II EAR antibodies are not preferentially presented by MHC complexes (Figure 8). By comparing B cell epitope with MHC epitope predictions on the 1^st^ degree classifiable peptides, both predictions (epitope binding to antibodies vs. to MHC complexes) seem to correlate to some degree with each other, even though different types of epitope interactions are described. Most likely, binding modes that favor EAR and binding modes involved in peptide binding to MHC class I and class II share basic principles of constraints, in particular in regard to EAR of classifiable peptides.

### Eliciting IgG Antibody Responses

Two modes of inducing antibody responses can be considered: (1) Type I EAR antibodies might have been induced by peptide presentation via MHC class II plus T cell receptor mediated induction of secondary signals that lead to costimulation of B cells. (2) Antibodies that recognize Type II EAR peptides might have evolved in a T cell-independent manner. As reviewed by Pone et al. [7], activation of the B cell receptor alone or in conjunction with Toll-like-receptor engagement can lead to polyclonal and antigen-specific immunoglobulin production and class switching independently of any T cell help. For instance, autoantigenic properties of hnRNPs (hnRNP-A/B antigens) appear to be mediated by associated nucleic acids binding to Toll-like receptors TLR7 and 9 [42]. Relating both types of EAR to existing assumptions and models describing humoral immune responses indicates that possibly Type I EAR might have been derived from T cell-dependent and Type II EAR from T cell independent antibody formation. In case both EAR types result from different modes of eliciting antibody responses, antibody structures specifying both EAR types might have been diverged over time in evolution and should by now differ from each other by their paratope structures.

### Physiology of IVIG

The existence of two distinct EAR modes on the epitope level might have counterparts on the corresponding paratope level of binding antibodies. The question is whether these two EAR groups can be related to specific types of antibody species and whether paratope binding rules can be established in the future as well. It will be instrumental to study in more detail more selected epitope-paratope interactions, e.g. by molecular modeling, to pinpoint subgroups with respect to binding modes and to determine the underlying structural (and maybe also dynamic) features. In particular, antibodies present in IVIG are capable of binding to a significant extent to continuous peptides of human origin, implying that antibodies circulating in the human body should under physiological conditions bind peptides floating around in the human blood stream. The physiological role of antibodies binding to circulating peptides can now be monitored by making use of IVIG specific peptide panels. During therapeutic application of IVIG, half-lives of human antibodies present in IVIG preparations might be determined in the circulation. Adverse reactions seen while administering IVIG preparations might be partly explained by instant formation of immune complexes due to specific antibody reactivities present in IVIG preparations. IVIG antibodies binding to specific peptides present in the circulation should have shorter half-lives than antibodies that do not find peptide binding partners. The IVIG data set established here might serve as a benchmark to explore the nature of antibody paratopes interacting with peptide/protein epitopes. Numerous sequencing efforts are currently undertaken to determine immunoglobulin repertoires of circulating human B cells [43]. In the near future, the presence of Type I and Type II EAR of epitope-paratope structures might be experimentally verified in conjunction with ongoing antibody repertoire analysis.

### Immunological Perspectives

Antibodies from healthy human donors have been found by epitope profiling of IVIG preparations to bind to a vast number of peptides of human origin. In absolute terms, the human immune system should not have any antibodies directed against their own proteome [44]. However, due to the way how antibodies are generated, sophisticated mechanisms have to be in place either to prevent or to minimize B cells from generating autoreactive antibodies [45] or to remove B cells (e.g. by autophagy) to eliminate the generation of highly reactive auto-antibodies [46], [47]. Possibly, the immunoglobulin locus including the machinery regulating humoral in conjunction with cellular immune responses has been modified over millions of years under constraints to select immunoglobulin structures harmless to their own organisms. Basic antibody structures and regulatory mechanisms might have been generated preventing the formation of highly reactive antibodies that bind to own peptide/protein structures. About 25% of peptides in our training and test sets were scored as “binding” to IVIG with high confidence. However, this percentage ignores the number of peptides tested in total and is probably overestimated. With respect to the 75,534 peptides of the full analysis set the percentage drops to 9.05%. This percentage might now be an underestimate: for higher confidence we ignored probably numerous potentially true positive (binding) peptides in the range below signal 10,000. Our EAR analysis leads to the hypothesis that under physiological conditions immunoglobulins possibly contribute to the homeostasis of the immune system [48] by constantly capturing circulating peptides that originate from human proteins. The postulated scavenger function of eliminating self-peptides should not lead to inflammatory processes as exemplified by autoimmune diseases.

#### Note

These data sets have been donated to the DREAM Challenge to encourage other immunoinformatic groups to test their algorithms, see http://wiki.c2b2.columbia.edu/dream/index.php/D5c1. A webserver for predicting EAR of peptide sequences is available at www.sysmed-immun.eu/EAR.

## Supporting Information

Table S1 **List of the peptides of the training set.** Information includes their origin, their signal intensities measured after staining with IVIG, their MHC binding predictions, their cumulative count of amino acids N, Q, E and Y, W, F, respectively, and their individual classifications according to the respective prediction. A detailed legend is given in a separate sheet of the Excel file.
(XLS)

Table S2 **List of the peptides of the test set. Information includes their origin, their cumulative count of amino acids N, Q, E and Y, W, F, respectively, and their individual classifications according to the respective prediction.** A detailed legend is given in a separate sheet of the Excel file.
(XLS)

Table S3 **Epitope propensity values for each amino acid computed for various groups of peptides selected according to their classifiability.** The table contains the numeric values plotted in Figures 4 and 5.
(XLS)

Table S4 **List of PDB identifiers with their computed enrichment in the epitope amino acids Y+W+F versus N+Q+E.**
(XLS)

File S1 **Background on IVIG.**
(PDF)

File S2 **Information on the analyzed peptide sets, the distribution of the signals and the reproducibility of the measurements; (1) origin of the peptides of the input data set, (2) signal intensity distributions in the basic and input data sets, (3) random splitting of the input data set: Procedure and reproducibility, (4) Reproducibility of peptide microarray staining with IVIG.**
(PDF)

File S3 **Detailed description of: (1) the state-of-the-art in epitope prediction and characterization, (2) the attributes for the employed machine learning approach, (3) the six-step tuning procedure of the machine learning approach, (4) the machine learning algorithms, (5) the performance measures of the epitope predictions and (6) the epitope-antibody-reactivity (EAR) rules.**
(PDF)

File S4 (1) Additional machine learning results: Confusion matrices of dividing peptides into classifiable and unclassifiable. (2) Additional PWM results: heat maps for the 1st degree classifiable and unclassifiable peptides, respectively; ROC curve for 2^nd^ degree unclassifiable peptides, now classified by PWM scores. (3) Details on MHC class I and class II prediction servers.
(PDF)
