# Supplementary material for: Epitope Predictions Indicate the Presence of Two Distinct Types of Epitope-Antibody-Reactivities Determined by Epitope Profiling of Intravenous Immunoglobulins
Source: PLoS One. 2013 Nov 11;8(11):e78605. doi: 10.1371/journal.pone.0078605 (PMC3823795; doi:10.1371/journal.pone.0078605)
Supplement: File S1 — Background on IVIG. (PDF) [file pone.0078605.s005.pdf]

Supporting information File S1

Background on Intravenous Immunoglobulin (IVIG)

Intravenous Immunoglobulin (IVIG) is usually prepared from the serum of between 1,000 and 15,000 donors per batch. It is used to treat neurological disorders including dermatomyositis, Guillain–Barre syndrome, chronic inflammatory demyelinating polyneuropathy (CIDP), multifocal motor neuropathy (MMN), myasthenia gravis and stiff person syndrome, as well as in immunology primary and secondary antibody deficiencies [1], [2]. The predominant mechanism how IVIG operates depends on the immunoglobulin dose and the pathogenesis of the disease [3]. Commercial IVIG preparations contain a 'species repertoire' of antibody specificities that are suspected to neutralize a wide range of antigens including pathogens and superantigens [4]. Significant batch-to-batch variation has been observed in the concentration of particular antibodies [5], [6]. Interestingly, IVIG seems to have a considerable inhibitory effect on mitogen induced T cell proliferation in vitro [7], most likely due to intact IgG, since less evidence exists for the role of Fc fragments alone [8]. Inhibitions of antigen-dependent and antigen-independent responses have been noted by IVIG in a dose-dependent manner [9]. As such, IVIG suppresses the proliferation of antigen-specific T cells without inducing apoptosis, while the cells remain refractory to induction of apoptosis by CD95 ligation [10]. The antibodies present in IVIG are of IgG nature, implying that these antibodies have been induced by T-cell help. IVIG preparations were used from commercial vendors such as Omrix, Israel; Sclavo Vena NIV, Italy; Tegeline, Laboratoire Français du Fractionnement et des Biotechnologies LFB, France; Octagam, Octapharma GmbH, Langenfeld, Germany; Intratect, Biotest Pharma GmbH, Dreieich, Germany.

Supporting information File S1 Table 1: Sample statistics on IVIG used for staining *

**Preparation**	**Input dataset**
mixed IVIG preparation	2,569
Biotest Intratect®	2,637
LFB Tégéline®	3,328
Octapharma Octagam®	2,722
Omrix Omr-IgG-am™	11,301
Sclavo Ig-Vena™	4,721
**Total**	**27,278**

*number of different peptides included in the input dataset, i.e. within the thresholds (signals ≤ 100 OR signals > 10,000), and stained with the indicated IVIG

References:

1. El-Shanawany T, Sewell WA, Misbah SA, Jolles S (2006) Current clinical uses of intravenous immunoglobulin. Clinical medicine 6: 356-359.
2. Kivity S, Katz U, Daniel N, Nussinovitch U, Papageorgiou N, et al. (2010) Evidence for the use of intravenous immunoglobulins--a review of the literature. Clinical reviews in allergy & immunology 38: 201-269.
3. Jolles S, Sewell WA, Misbah SA (2005) Clinical uses of intravenous immunoglobulin. Clinical and experimental immunology 142: 1-11.
4. Takei S, Arora YK, Walker SM (1993) Intravenous immunoglobulin contains specific antibodies inhibitory to activation of T cells by staphylococcal toxin superantigens [see comment]. The Journal of clinical investigation 91: 602-607.
5. Lamari F, Anastassiou ED, Tsegenidis T, Dimitracopoulos G, Karamanos NK (1999) An enzyme immunoassay to determine the levels of specific antibodies toward bacterial surface antigens in human immunoglobulin preparations and blood serum. Journal of pharmaceutical and biomedical analysis 20: 913-920.
6. Svetlicky N, Ortega-Hernandez OD, Mouthon L, Guillevin L, Thiesen HJ, et al. (2013) The advantage of specific intravenous immunoglobulin (sIVIG) on regular IVIG: experience of the last decade. Journal of clinical immunology 33 Suppl 1: S27-32.
7. Kawada K, Terasaki PI (1987) Evidence for immunosuppression by high-dose gammaglobulin. Experimental hematology 15: 133-136.
8. Klaesson S, Ringden O, Markling L, Remberger M, Lundkvist I (1993) Immune modulatory effects of immunoglobulins on cell-mediated immune responses in vitro. Scandinavian journal of immunology 38: 477-484.
9. van Schaik IN, Lundkvist I, Vermeulen M, Brand A (1992) Polyvalent immunoglobulin for intravenous use interferes with cell proliferation in vitro. Journal of clinical immunology 12: 325-334.
10. Aktas O, Waiczies S, Grieger U, Wendling U, Zschenderlein R, et al. (2001) Polyspecific immunoglobulins (IVIg) suppress proliferation of human (auto)antigen-specific T cells without inducing apoptosis. Journal of neuroimmunology 114: 160-167.
