# Supplementary material for: Epitope Predictions Indicate the Presence of Two Distinct Types of Epitope-Antibody-Reactivities Determined by Epitope Profiling of Intravenous Immunoglobulins
Source: PLoS One. 2013 Nov 11;8(11):e78605. doi: 10.1371/journal.pone.0078605 (PMC3823795; doi:10.1371/journal.pone.0078605)
Supplement: File S2 — Information on the analyzed peptide sets, the distribution of the signals and the reproducibility of the measurements; (1) origin of the peptides of the input data set, (2) signal intensity distributions in the basic and input data sets, (3) random splitting of the input data set: Procedure and reproducibility, (4) Reproducibility of peptide microarray staining with IVIG. (PDF) [file pone.0078605.s006.pdf]

Supporting information File S2

Information on the analyzed peptide sets, the distribution of the signals and the reproducibility of the measurements

1. Origin of the peptides of the input dataset

File S2 Table 1 summarizes the extended tables S1 and S2 which give the origin and nature of each peptide individually.

File S2 Table 1: Statistics of the origin and nature of peptide sequences used *

	**Training set**				**Test set**		**Input dataset**	
	**"binding"**		**"non-binding"**					
**Human** [a]	2,373	69.4 %	7,759	75.9 %	10,027	73.5 %	**20,159**	**73.9 %**
**Random** [b]	755	22.1 %	1,475	14.4 %	2,196	16.1 %	**4,426**	**16.2 %**
**Mutation** [c]	101	3.0 %	519	5.1 %	704	5.2 %	**1,324**	**4.9 %**
**Neo** [d]	165	4.8 %	359	3.5 %	569	4.2 %	**1,093**	**4.0 %**
**Citrulline** [e]	3	0.1 %	70	0.7 %	71	0.5 %	**144**	**0.5 %**
**Other** [f]	23	0.7 %	36	0.4 %	73	0.5 %	**132**	**0.5 %**
**Total**	**3,420**		**10,218**		**13,640**		**27,278**	

* The left subcolumn of each column shows the absolute number of peptides and the right subcolumn the percentage of peptides in the respective group.

[a] derived from human protein

[b] random sequence, not derived from any known protein

[c] peptide with amino acid exchange compared to database sequence that has been mutated intentionally for a different project

[d] derived from a sequence related to pathophysiology (usually tumor neo-antigen)

[e] peptide with citrulline replacing an initial arginine residue

[f] not assigned

2. Signal intensity distributions in the basic and input datasets

Our machine learning approach requires a faithful assignment of peptides to the classes "binding" and "non-binding". Therefore, our goal was to assure this requirement rather than include the highest possible peptide number and run the risk to include wrongly assigned peptides.

Part of the reason for the chosen thresholds is based on our experience with thousands of different peptide arrays in different projects (in total, 20 million single/60 million in triplicate EAR measures). The thresholds of peptides measured in triplicate on each chip with values below 100 and above 10,000 (after background subtraction) are quite robust, see below.

File S2 Figures 1 and 2 illustrate the distributions of the signal intensities measured on all peptides of the basic input set and the training set, respectively. The figures show that the thresholds of peptides designated "non-binding" (signal intensities ≤100) and "binding" (signal intensities > 10,000) are many standard deviations apart from each other. Thus, the assignment of these groups to "non-binding" and "binding" can be considered to be very robust. To exclude peptides that run the risk of being wrongly assigned to both groups, peptide with signals in between 100 and 10 000 were omitted from the analysis.

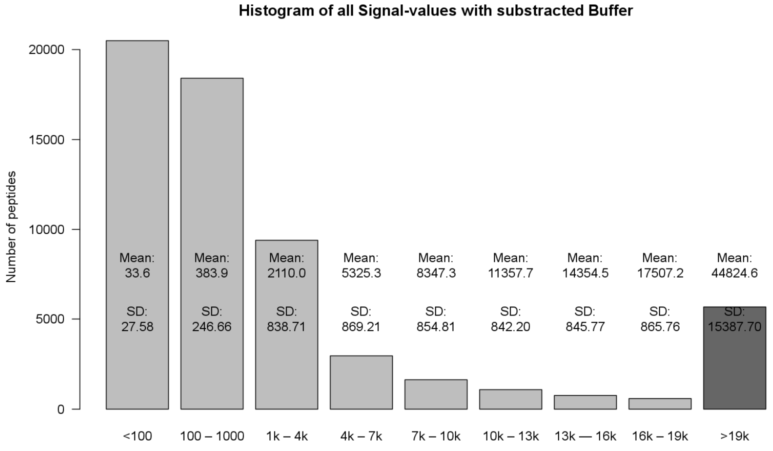

File S2 Figure 1: Signal intensity distribution of the basic peptide set. Histogram of signal intensity bins as indicated along with the arithmetic mean and the standard deviation (SD) for each bin. Note, that for each peptide, background values of secondary antibody only-signals have been subtracted.

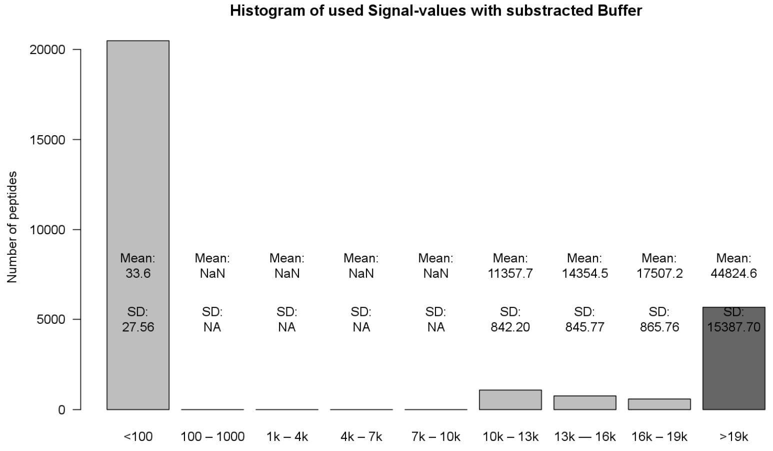

File S2 Figure 2: Signal intensity distribution of the input data set. Histogram of signal intensity bins as indicated along with the arithmetic mean and the standard deviation (SD) for each bin. Note, that for each peptide, background values of secondary antibody only-signals have been subtracted. Here, only the bins that contain the peptides of the input data set (that was then split into training and test set) are indicated.

3. Random splitting of the input dataset: Procedure and reproducibility of results

Splitting of the input data set into training and test sets were done randomly. However, peptides from the "binders" and "non-binder" group were shuffled individually and then split in half. The following R code has been used:

```
loTraining = sample(loPeptides, round(length(loPeptides)/2))
hiTraining = sample(hiPeptides, round(length(hiPeptides)/2))
testset = setdiff(sample(c(loPeptides, hiPeptides)), c(loTraining, hiTraining))
```

To demonstrate that the overall data stays the same, PWM-generation (**File S2 Table 2**) and ROC of prediction were repeated 10 times. Spearman correlation coefficients between all 10 generated PWMs were all larger than 0.95. Thus, the PWMs were very similar to each other independent of the split dataset used. Further, the ROC curves of the 10 predictions with the different split datasets were overlaying each other with almost identical AUCs. (Supporting information file Sa Figure 3). Altogether these data argue that our results were reproducible and not biased by the initial random splitting of the input dataset.

File S2 Table 2: Reproducibility of PMW generation after random splitting of the input dataset into training and test set *.

	PWM1	PWM2	PWM3	PWM4	PWM5	PWM6	PWM7	PWM8	PWM9	PWM0
**PWM1**	1	0,968	0,970	0,963	0,961	0,967	0,965	0,969	0,960	0,971
**PWM2**	0,968	1	0,970	0,963	0,969	0,970	0,969	0,972	0,962	0,973
**PWM3**	0,970	0,970	1	0,966	0,972	0,968	0,970	0,971	0,963	0,973
**PWM4**	0,963	0,963	0,966	1	0,961	0,965	0,965	0,965	0,961	0,970
**PWM5**	0,961	0,969	0,972	0,961	1	0,968	0,966	0,968	0,968	0,970
**PWM6**	0,967	0,970	0,968	0,965	0,968	1	0,963	0,969	0,960	0,972
**PWM7**	0,965	0,969	0,970	0,965	0,966	0,963	1	0,968	0,968	0,974
**PWM8**	0,969	0,972	0,971	0,965	0,968	0,969	0,968	1	0,965	0,969
**PWM9**	0,960	0,962	0,963	0,961	0,968	0,960	0,968	0,965	1	0,964
**PWM0**	0,971	0,973	0,973	0,970	0,970	0,972	0,974	0,969	0,964	1

* Pearson correlation coefficients of ten different ratio PWMs on the training set, obtained after random splitting of the input dataset. The ratio PMW were calculated using the ratio of the frequency of the occurrence of a given amino acid at a given peptide position in "binding" versus the "non-binding" peptides.

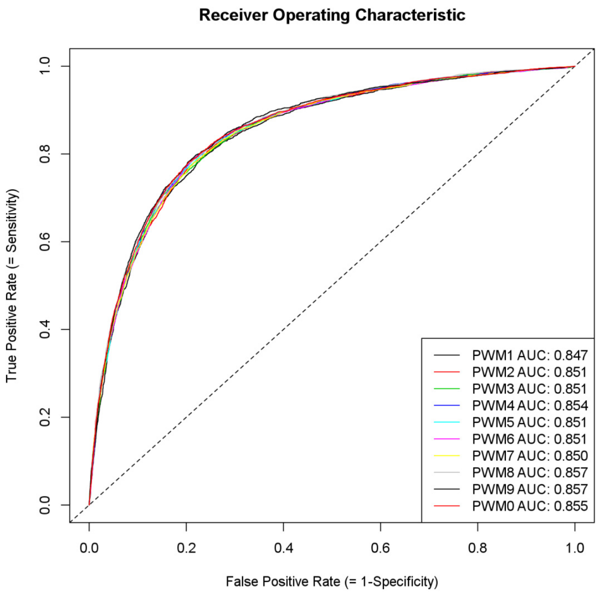

File S2 Figure 3: Performance comparisons of the predictions for IVIG binding when PMW were built on different trainings sets after random splitting of the basis data set.

4. Reproducibility of peptide microarray staining with IVIG

We used a threshold approach to to assign the peptides to the group "binding" or "non-binding" with respect to IVIG antibodies. Because the two groups were well separated (binding means signals > 10,000 while non-binding ≤ 100), these assignments were very robust. The absolute signal intensities beyond a threshold do not matter for the assignment and thus the analysis in this manuscript, e.g. if a peptide had signals of 12,000 or 57,000 would not have any impact.
Since the peptide content differs from microarray lot to lot, a different number of measurements was obtained for different peptides (the range was 2 to 72). We chose to always use the highest signal that occurred among all experiments for a particular peptide.

The **File S2 Table 3** below shows measurements for 8 peptides (4 each for the > 10,000 OR ≤ 100 threshold group) in 10 different microarray experiments and illustrates the robustness of the assignments.

In addition, **File S2 Figure 3** (next page) shows scatter plots of all peptide signals of a certain microarray lot when stained with different IVIG samples. The graphs demonstrate that peptides that show high signals for one IVIG in general also display such reactivities when the experiment was repeated or when a different IVIG was used. Thus, the assigments to "binding" and "non-binding" looked robust.

File S2 Table 3: Reproducibility of repeated measurements for selected peptides assigned to the groups "non-binding" or "binding" with respect to IVIG

Peptides	Meas1 [a]	Meas2 [a]	Meas3 [a]	Meas4 [a]	Meas5 [a]	Meas6 [a]	Meas7 [a]	Meas8 [a]	Meas9 [a]	Meas10[a]
IDDRC...	53	18	44	21	37	16	59	33	46	14
FKWLK...	29	25	61	28	50	21	16	46	32	17
QHRIL...	14	31	20	10	15	25	9	18	4	21
GQVRT...	26	23	3	21	16	9	16	11	9	19
RILAK...	60923	59060	60067	58074	40622	49477	33552	21820	23427	1386
LMSAT...	62504	60944	55981	8583	58963	41551	26418	47970	50729	407
IDFHY...	62766	60422	55546	11214	58772	40357	31090	46622	52790	532
REGGL...	61077	57368	59340	16788	48703	20146	39008	30219	49171	1273

[a] Measurements (Meas) 1-10: Indicated values represent the signal intensities (the mean of triplicate measurements on the same chip after subtraction of background values for secondary antibody alone).

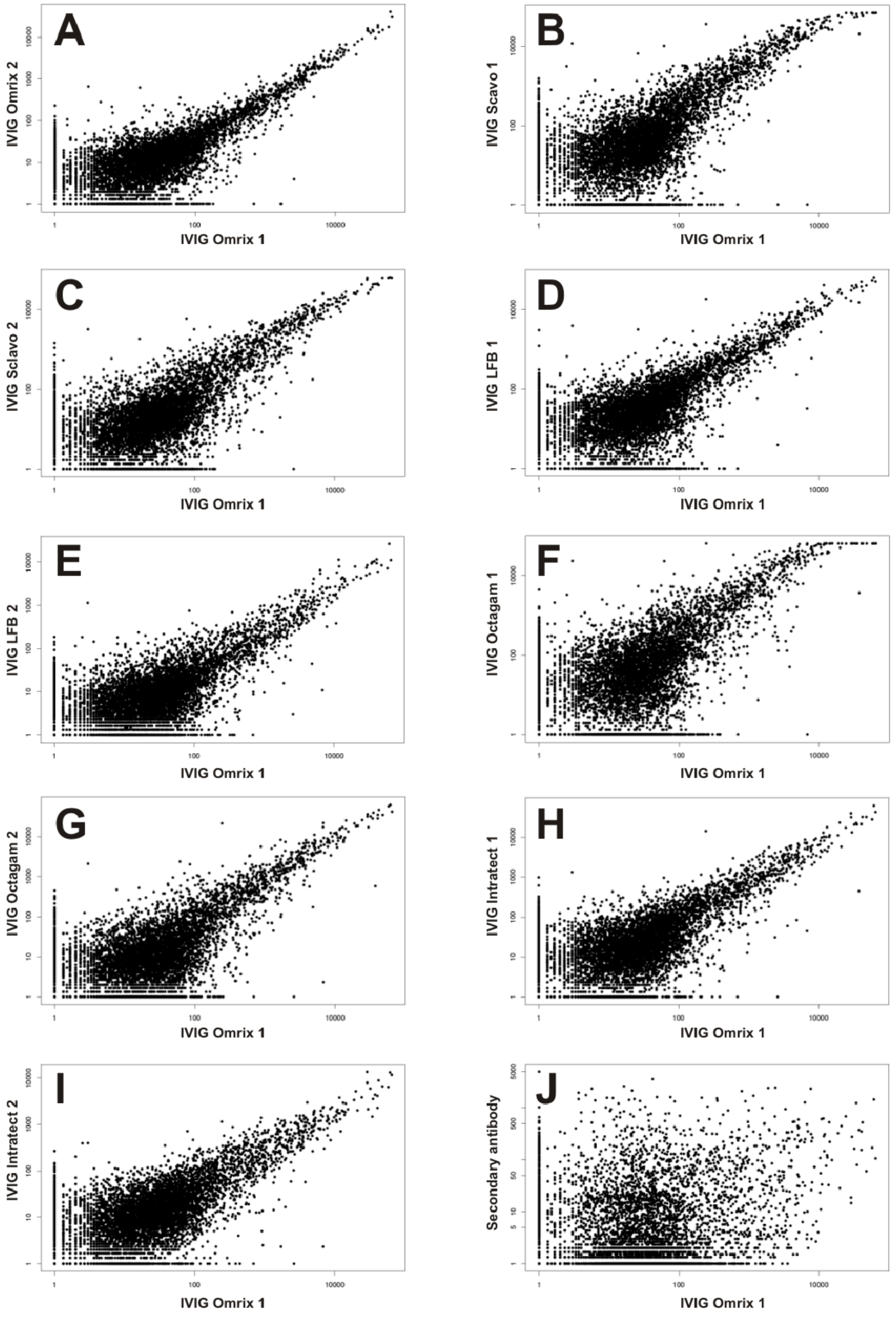

File S2 Figure 3: Scatter graphs to illustrate the reproducibility of staining between different IVIG samples. Panels A-I: Comparison of one IVIG against another IVIG specified by the name of the supplier. Note that for each of the 5 IVIG samples a replicate was made (distinguished by IVIG name and affix 1 or 2). In panel A the replicates for IVIG Omrix are directly compared (technical replicate with the same Omrix sample). Panel J: Scatter plot for IVIG Omrix against the control, a chip stained with secondary antibody only (y-axis has a different scale !). This plot shows no correlation. The plots are in logarithmic scale.
