# Supplementary material for: Epitope Predictions Indicate the Presence of Two Distinct Types of Epitope-Antibody-Reactivities Determined by Epitope Profiling of Intravenous Immunoglobulins
Source: PLoS One. 2013 Nov 11;8(11):e78605. doi: 10.1371/journal.pone.0078605 (PMC3823795; doi:10.1371/journal.pone.0078605)
Supplement: File S3 — Detailed description of: (1) the state-of-the-art in epitope prediction and characterization, (2) the attributes for the employed machine learning approach, (3) the six-step tuning procedure of the machine learning approach, (4) the machine learning algorithms, (5) the performance measures of the epitope predictions and (6) the epitope-antibody-reactivity (EAR) rules. (PDF) [file pone.0078605.s007.pdf]

Supporting information File S3

1. State of the art in epitope prediction and characterization

The first attempts to predict continuous B-cell epitopes were based on propensity scales. These scales assign each of the 20 standard amino acids a value associated with some physico-chemical property. In the most simple epitope prediction approach, an average propensity score within a sliding window was computed for each amino acid in a protein, and those amino acids whose scores exceeded a given threshold were considered parts of an epitope. Numerous propensity scales were used: flexibility [1], solvent accessibility [2], hydrophobicity [3], antigenicity, which is simply the occurrence in known epitopes [4], the occurrence in turns of proteins [5] and others. However, a survey of 484 propensity scales by Blythe & Flower [6] showed that even the best of them are only marginally better at epitope prediction than random. Epitope prediction with a combination of amino-acid propensity scales proved more successful than with single scales. Pellequer & Westhof [7] developed a program called PREDITOP, which used 22 propensity scales for epitope prediction. Alix [8] used several propensity scales related to protein secondary structure, hydrophobicity, solvent accessibility and flexibility in his program PEOPLE. Odorico & Pellequer [9] used even more propensity scores, over 30, in their program BEBITOPE. Finally, Saha & Raghava [10] developed a web server for epitope prediction called BcePred, which combined seven propensity scales.

The attempts to predict epitopes also resulted in the characterization of the properties of the epitopes. One such property is the flexibility of the side chain of the constituent amino acids [11], which makes them easier to adjust to the antibody [12]. Another is the tendency to appear on the surface of antigens [13], where they are accessible to antibodies. Surface amino acids are often hydrophilic, while the ones in the interior of proteins are more often hydrophobic, so epitopes are characterized by low hydrophobicity [14]. The amino acids that often occur in turns of proteins are common in epitopes because such amino acids are both flexible and accessible [15]. It was observed that epitopes are enriched in tyrosine and tryptophan, and to have preference for polar amino acids [11].

2 Attributes for machine learning

There are eight attribute vectors that have been used to represent a peptide.

Frequency: This attribute vector consists of the frequencies of the amino acids in the peptide. 22 amino acids are present in the data set: the 20 standard ones, Z (which stands for citrulline) and X (which stands for an unknown amino acid). The peptide is split into p parts of equal length (or as close to equal as possible), and the frequencies are computed for each part separately. The attribute vector thus has the form:

$[A_1, C_1, ..., Z_1; ...; A_p, C_p, ..., Z_p]$.

A_i is the frequency of the amino acid A in the i-th part of the peptide. The best value of p as determined in the fourth step of parameter tuning was $p = 3$.

Frequency difference: This attribute vector consists of the differences between the frequencies of the amino acids in the peptide. While the information about such differences is already contained in the frequency attribute vector, there may be an advantage of providing it explicitly. For example, let the difference between frequencies $A - C > 0.3$ indicate that the peptide contains an epitope. A machine learning algorithm may miss this pattern if it is based on different individual frequencies A and C (such as $A = 0.8$, $C = 0.3$; $A = 0.6$, C=0.2; etc.), because each of them occurs rarely and consequently does not seem important. The peptide is again split into p equal parts, and the differences are computed for each part separately. Frequency differences turned out to perform better in combinations with frequencies themselves, so plain frequencies were added to the attribute vector. Its form is thus:

$[A–C_1, ..., A–Z_1, ..., Z–Z_1; ...; A–C_p, ..., A–Z_p, ..., X–Z_p; A, ..., Z]$.

$A–C_i$ is the difference between the frequencies of the amino acids A and C in the i-th part of the peptide, and A is the frequency of the amino acid A over the whole peptide. The best value of p was $p = 2$.

Subsequence frequency: This attribute vector consists of the frequencies of the subsequences of the peptide of lengths up to l. Since few longer subsequences occur often, which makes it difficult for a machine learning algorithm to recognize them as important, the definition of a subsequence is relaxed by allowing gaps of total length up to g. A subsequence with gaps does not count as a whole subsequence, but only as w^g of a subsequence, where $w \leq 1$. To keep the number of attributes under control, only the first c most common subsequences of each length in the training data are included. The attribute vector thus has the form:

$[S_{11}, S_{12}, ..., S_{1c}; S_{21}, S_{22}, ..., S_{2c}; ...; S_{l1}, S_{l2}, ..., S_{lc}]$.

S_{ij} is the frequency of the j-th most common subsequence of length i in the peptide. The best values of the parameters were $l = 5$, $g = 0$ and $c = 25$.

This attribute vector plays a role similar to the subsequence kernel used by EL-Manzalawy et al. [16], [17]. It performed better then the subsequence kernel on the training set, though – possibly because the subsequence kernel takes into account too many subsequences, including misleading ones. It should be noted that because this attribute vector consists of the most common subsequences, its exact composition depends not only on its parameter values, but on the training set as well.

Amino-acid property: This attribute vector consists of the average values of 19 amino-acid side-chain properties. The peptide is split into p equal parts and the properties are averaged over each part separately. The following properties are included:

- Acidity: the acidity equals pK_a – 14 for acids, pK_a + 2 for bases and 0 for neutral. The values of pK_a [18] range from –2 to 14, are small for strong acids and weak bases, and large for weak acids and strong bases (the range is valid for acids and bases in general, not specifically for amino acids). The scale was thus chosen so that the values for acids are between –16 (strongest) and 0 (weakest), and the values for bases between 0 (weakest) and 16 (strongest).
- Accessibility: the accessibility to solvent, averaged over the values given by Janin et al. [19] and Bordo & Argos [20].
- Antigenicity: the frequency in known epitopes, divided by the frequency in non-epitopes [4].
- Aromaticity: 1 for aromatic amino acids and 0 for the rest [18].
- Composition: atomic weight ratio of hetero (non-carbon) elements in end groups or rings to carbons in the side chain [21].
- Flexibility: flexibility as measured by atomic displacement in X-ray crystallography [22].
- Hydrogen bonding: 1 for the amino acids that form hydrogen bonds and 0 for the rest [23].
- Hydrophobicity: hydrophobicity as measured by high-performance liquid chromatography [3].
- Occurrence in turns of proteins [5].
- Polarity: polarity given by Grantham [21].
- Preference for α-helices, β-sheets and reverse turns [24].
- Size: van der Waals volume [18].
- Summary factors 1–5: five factors best summarizing 494 amino-acid properties, obtained by multivariate statistical analysis [25].

Plain amino-acid frequencies were added to the attribute vector, resulting in:

$[acidity_1, accessibility_1, ..., factor5_1; ...; acidity_p, accessibility_p, ..., factor5_p; A, ..., Z]$.

The value $acidity_i$ is the average acidity in the i-th part of the peptide etc., and A is the frequency of the amino acid A over the whole peptide. All 19 properties turned out to be valuable, as removing any of them resulted in worse classification. The best value of p was $p = 2$.

Class frequency: This attribute vector consists of the frequencies of classes of amino acids in the peptide. For example, such a frequency is the frequency of *acidic* amino acids. The classes of amino acids are based on the 19 properties described under the amino-acid-property attribute vector are included. This results in 17 ways to classify amino acids, as the preferences for α-helices, β-sheets and reverse turns are merged. The peptide is split into p equal parts and the class frequencies computed for each part separately. The following ways to classify amino acids are included:

- Acidity: acidic (ED), basic (HKR), neutral (ACFGILMNOPQSTVWYXZ)
- Accessibility: low (CIFVLGAMW), medium (XZHSTY), high (PDNQERK)

- Antigenicity: low (NMEDRGW), medium (TKSQXZAPF), high (HIYLVC)
- Aromaticity: yes (FHWY), no (ACDEGKMNPQRSTILVXZ)
- Composition: zero (AFILMV), low (WYKPHXZRTGQE), high (NDSC)
- Flexibility: low (WYFCIVH), medium (LMAGTXZ), high (RSNQDPEK)
- Hydrogen bonding: yes (DEHKNQRSTWYXZ), no (ACFGILMPV)
- Hydrophobicity: low (DENSQGKTR), medium (AHPCXZ), high (YVMILFW).
- Occurrence in turns: low (IMLV), medium (TYFAQKCXZRWE), high (HPNGDS)
- Polarity: non-polar (LIFWCMVYPAGXZ), polar neutral (TSHQN), polar positive (RK), polar negative (ED)
- Preference for secondary structures: α-helices (ACLMEQHK), β-sheets (VIFYWT), reverse turns (GSDNP), indifferent (RXZ)
- Size: tiny (GAS), small (CPDTNV), large (EXZQHILMFKYRW)
- Summary factor 1: low (CVILF), medium (MWAGSTXZPYH), high (QNDERK)
- Summary factor 2: low (MEAL), medium (FKIHVQRXZWDTC), high (NYSGP)
- Summary factor 3: low (SDQHPLCAV), high (XZKWNGERFITMY)
- Summary factor 4: low (WHCMY), medium (QFKDNXZEIPR), high (STGVLA)
- Summary factor 5: low (DSQPVLE), medium (CWAHXZF), high (INMTYKGR)

Plain amino-acid frequencies are added to the attribute vector, resulting in:

[$freq$(acidity)$_1$, ..., $freq$(factor5)$_1$; ...; $freq$(acidity)$_p$, ..., $freq$(factor5)$_p$; A, ..., Z], where
$freq$(acidity)$_i$ = [$acidic_i$, $basic_i$, $neutral_i$]
...
$freq$(factor5)$_i$ = [$factor5\text{-}low_i$, $factor5\text{-}medium_i$, $factor5\text{-}high_i$].

The value $acidic_i$ is the frequency of acidic in the i-th part of the peptide etc., and A is the frequency of the amino acid A over the whole peptide. The best value of p was $p = 3$.

Class subsequence frequency: This attribute vector is similar to the subsequence-frequency vector. The difference is that instead of individual amino acids, the subsequences consist of the 17 classes of amino acids described under the class-frequency attribute vector. For example, the subsequence of amino acids “EADC” is replaced with “anan”, where “a” stands for acidic and “n” for neutral. This addresses the already mentioned problem of few subsequences occurring often and thus being recognized as important. The problem is addressed by replacing 22 individual amino acids with three classes, resulting in fewer different subsequences and thus greater recurrence of them. A subsequence can again have up to g gaps and has a weight of w^g. Only the first c most common subsequences of each length in the training data are included. Plain amino-acid frequencies were added to the attribute vector, resulting in:

[$subseq$(acidity), ..., $subseq$(factor5); S_{11}, ..., S_{1c}; ...; S_{l1}, S_{l2}, ..., S_{lc}], where
$subseq$(acidity) = [$acidity_{11}$, ..., $acidity_{1c}$; ...; $acidity_{l1}$, ..., $acidity_{lc}$]
...
$subseq$(factor5) = [$factor5_{11}$, ..., $factor5_{1c}$; ...; $factor5_{l1}$, ..., $factor5_{lc}$].

The value $acidity_{ij}$ is the frequency of the j-th most common class subsequence of length i in the peptide when amino acids are classified by acidity (into acidic, basic, neutral). S_{ij} is the frequency of the j-th most common amino-acid subsequence of length i. The best values of the parameters were $l = 2$, $g = 5$, $w = 0.5$ and $c = 25$.

Pair: This attribute vector consists of the frequencies of pairs of amino acids with a certain distance between them. For example, such a frequency is the frequency of the pair (A, C) with distance 3. The rationale is that epitopes might bind antibodies at more than one anchor position due to the shape of the paratope, the antigen-binding site on the antibody. The number of such attributes is very large, so two measures can be taken to reduce it. First, s distances can be merged into one. If $s = 3$, an example attribute is the frequency of the pair (A, C) with the distance 4, 5 or 6 between the members of the pair. Second, one or both of the amino acids in each pair can be replaced with a class of amino acids. If one of the amino acids is replaced with acidity class and $s = 3$, an example attribute is the frequency of the pair (A, acidic) with the distance 4, 5 or 6 between the members. The attribute vector thus has the form:

$[pair(A_1, A_2), ..., pair(A_1, A_{n2}); ...; pair(A_{n1}, A_1), ..., pair(A_{n1}, A_{n2})]$, where
$pair(A_i, A_j) = [(A_i, A_j)$ with $d_1, ..., (A_i, A_j)$ with $d_{\max}]$.

A_i is the i-th amino acid or class of amino acids, $n1$ and $n2$ are the numbers of different amino acids or classes for the first and second member of the pair, d_k is the k-th distance or a group of distances between the two amino acids (or classes) in the pair, and $d_{\max}$ is the maximum distance. The best classification was achieved with the first member of the pair being amino acid and the second aromaticity class. The best value of s was $s = 5$.

Fixed pair: This attribute vector consists of the frequencies of amino acids at a certain distance from the first position in the peptide, and the amino acid at the first position. For example, such a frequency is the frequency of the amino acid A at distance 3 from the first position. This attribute vector is similar to the pair vector, except that one member of the pair is fixed to the first position. The rationale is that when a peptide is placed on an array, the first (uppermost) position is the most exposed and the most likely to bind an antibody. However, EAR detection should not rely on unwanted binding particularities at the other end of fixed linear peptides. By ignoring the latter peculiarity, physically the eighth position within a 15mer peptide constitutes the central position for antibody recognition. Thus, if the antibody binding site does use the eight position as anchor position at its center. antibodies have enough antigenic space to bind the epitope coming from both ends. Again s distances can be merged and the amino acid can be replaced with a class of amino acids. The attribute vector thus has the form:

$[A_1$ at $d_1, ..., A_1$ at $d_{\max}; ...; A_n$ at $d_1, ..., A_n$ at $d_{\max}; first]$.

A_i is the i-th amino acid or class of amino acids, n is the number of different amino acids or classes, d_j is the i-th distance or a group of distances from the first position, $d_{\max}$ is the maximum distance, and $first$ is the first amino acid in the peptide. The best classification was achieved with amino acids (not classes) and $s = 5$.

3 Parameter tuning

Searching the whole space of the available options for the ML-advanced machine learning approach was infeasible, so we used a six-step tuning procedure. All tuning was done on the training set.

- In the first step we compared 41 machine learning algorithms from the Weka machine learning suite [26] on attribute vectors consisting of the frequencies of amino acids in the peptide. Default parameter values were used for the algorithms. The comparison resulted in a short list of eight algorithms with SVM [27] in the lead.
- In the second step we determined the provisional best attribute parameter values for each of the eight attribute vectors, using SVM.
- In the third step we compared the eight machine learning algorithms from the first step on each of the eight attribute vectors with the provisional best attribute parameter values as determined in the second step. Three of the algorithms performed best on at least one of the attribute vectors: SVM, logistic regression [28] and classification via regression [29].
- In the fourth step we compared the whole range of reasonable attribute parameter values using these three algorithms. Each algorithm was tested by itself, all three were combined by stacking and finally just the better two (SVM and logistic regression) were combined by stacking. The result of this step was the best attribute parameter values and the best machine learning algorithm for each of the eight attribute vectors.
- In the fifth step we tuned the parameter values of the machine learning algorithms selected in the previous step. The parameters were tuned separately for each of the eight attribute vectors.
- In the sixth step, we selected the best machine algorithm for the meta-classifier and tuned its parameters. 41 algorithms from the Weka machine learning suite were compared again.

The number of all possible parameter value combinations was in many cases still too large to search exhaustively, so we used an informal greedy search. We optimized the parameters one by one in what we deemed to be decreasing order of importance. For the parameters with few possible discrete values, all the values were tried. For the parameters with many discrete or continuous values, a few evenly spaced values were tried first. After that, additional more densely spaced values were tried in the most promising interval. The data set used during the first five steps was balanced using random undersampling (see the Data subsection). This was also the case for most of the sixth step. The best five candidate machine learning algorithms for the meta-classifier, however, were compared on the training set balanced with random oversampling, as well as on the original (imbalanced) training set. This was done so that at least in the last step the conditions for comparison were identical to the conditions under which the final classifier for epitope prediction was trained.

4 Machine learning algorithms

We describe only the most relevant machine learning algorithms. The three that were found most suitable to train the base classifiers were SVM, logistic regression and stacking. The meta classifier was trained by Pace Regression [30], which is a version of linear regression whose mathematical details are beyond this paper. The algorithm for the induction of human-readable rules, which was used for epitope analysis, is RIPPER. All algorithms are implemented in the Weka machine learning suite [26].

SVM: Attribute vectors of length L representing the peptides can be said to occupy an L-dimensional space. The SVM finds a hyperplane across this L-dimensional space that best separates the two classes. To deal with cases where the classes are not linearly separable, some instances of each class are permitted to be on the wrong side of the hyperplane. Such errors also allow for a greater margin between other instances and the hyperplane, which improves the generalization of the classifier, because small margins on the training data may result in misclassification on the slightly different test data. The trade-off between errors on the training data and forcing narrow margins is controlled by the complexity parameter C: increasing the value of C increases the cost of misclassification on the training data.

Another way to deal with classes that are not linearly separable is to map the attribute vectors into a higher-dimensional space. Let x_i and x_j be a pair of L-dimensional attribute vectors. Let $\Phi(x_i)$ and $\Phi(x_j)$ be these vectors mapped into a higher-dimensional space. Since computing the hyperplane that separates the classes only involves computing inner products of the attribute vectors, the mapping can be efficiently accomplished by a kernel function $K(x_i, x_j)$, which returns the inner product of $\Phi(x_i)$ and $\Phi(x_j)$ without explicitly performing the mapping. The linear kernel function returns the plain dot product: $K_{\mathrm{lin}}(x_i, x_j) = x_i \cdot x_j$. Linear kernel is just a special case of polynomial kernel: $K_{\mathrm{poly}}(x_i, x_j) = (x_i \cdot x_j)^p$. The PUK kernel [31] is based on the Pearson VII function, defined as follows:

$$K(x_i, x_j)_{\mathrm{PUK}} = \frac{1}{\left(1 + \left(\frac{2\sqrt{(x_i - x_j)^2}\sqrt{2^{1/\omega} - 1}}{\sigma}\right)^2\right)^{\omega}}.$$

The SVM does not return proper probabilities that an instance belongs to the class into which it is classified. Therefore, the values returned by the SVM are mapped to probabilities by logistic regression [32]. We used the Sequential Minimal Optimization (SMO) implementation of SVM [27], which is capable of performing the mapping. The SVM alone was used to train three of the base classifiers (see the list below). Four of the base classifiers used stacking, which also included SVM. The best parameter values as determined in the fifth step of the parameter tuning were as follows:

- Frequency: $C = 1$, PUK kernel with $\omega = 0.5$, $\sigma = 2.5$
- Subsequence frequency: $C = 1$, polynomial kernel with $p = 2$

- Amino-acid property: stacking, C = 2, PUK kernel with $\omega = 1$, $\sigma = 2$
- Class frequency: stacking, $C = 1$, linear kernel
- Class subsequence frequency: stacking, $C = 0.5$, linear kernel
- Pair: stacking, $C = 0.1$, polynomial kernel with $p = 2$
- Fixed pair: $C = 5$, linear kernel

Logistic regression: The logistic function $f(z)$ maps an input variable z to a value between 0 and 1. The input variable is a weighted sum of the attributes in the attribute vector $x = [x_1, x_2, ..., x_L]$, and the output is the probability that the peptide represented by the attribute vector x is positive. The function is defined as follows:

$f(z) = (1 + e^{-z})^{-1}$, where
$z = \beta_0 + \beta_1 x_1 + \beta_2 x_2 + ... + \beta_L x_L$.

To classify peptides using logistic regression [28], the parameters β_0 $\beta_1, ..., \beta_L$ must be estimated from the training data. If the number of attributes is large or if they are correlated, the estimates may be unstable. This can be mitigated by restricting the difference between successive parameters β_i, β_{i+1}. How much the parameters are restricted depends on the ridge parameter λ: increasing λ increases the restriction.

Logistic regression alone was used to train one of the base classifiers. Four of the base classifiers used stacking, which also included logistic regression. The best parameter values were as follows:

- Frequency difference: $\lambda = 10^{-8}$
- Amino-acid property: stacking, $\lambda = 0.1$
- Class frequency: stacking, $\lambda = 1$
- Class subsequence frequency: stacking, $\lambda = 50$
- Pair: stacking, $\lambda = 200$

Logistic regression was also used to divide peptides into classifiable and unclassifiable, with $\lambda = 0.1$.

Stacking: This method [33] is similar to our overall machine learning procedure. It uses a number of base classifiers, which are trained by different machine learning algorithms, but on the same attribute vector. Each of these base classifiers returns the probability that the peptide represented by the attribute vector contains an epitope. These probabilities form a new attribute vector, whose class is 1 if the peptide contains an epitope and 0 otherwise. A meta classifier, which uses regression, is then trained on the new attribute vectors to compute the final probability that the peptide contains an epitope. Stacking uses internal cross-validation: base classifiers are trained on a part of the data and used to classify the rest, which is then used to train the meta classifier.

We used the StackingC implementation [34] of stacking with five-fold internal cross-validation and linear regression to train the meta classifier. The base classifiers were

trained by SVM and logistic regression. Other machine learning algorithms we tried had issues with randomly oversampled data (see the Data subsection): because of the presence of the same peptides in training and test data during the internal cross-validation, their performance was evaluated too optimistically. As a consequence, they were assigned incorrect weights in the liner combination of the outputs of the base classifiers that constituted the meta classifier. Stacking was used in the base classifiers trained on four of the attribute vectors (note that the base classifiers in the overall machine learning procedure are meant here, not the base classifiers in stacking): amino-acid property, class frequency, class subsequence frequency and pair.

RIPPER: The name stands for Repeated Incremental Pruning to Produce Error Reduction [35], a machine learning algorithm that learns a set of rules to classify the data. The algorithm splits training data into a growing set to “grow” rules and a pruning set to “prune” them.

Growing a rule consists of repeatedly selecting the attribute with the highest information gain, which means that knowing its values gives the most information about the class, and adding it to the antecedent of the rule. This is repeated until all the instances matched by the antecedent of that rule belong to the same class.

No freshly grown rule misclassifies on the growing set, but the question is how well it does on previously unseen data. An example on such data is the pruning set, so each rule is tested on the pruning set. Each of the conditions in the rule is considered for removal. Those whose removal improves the performance on the pruning set are in fact removed.

After growing and pruning each rule, the instances matched by the antecedent of that rule are deleted from the growing set. The procedure is repeated until the rule added last increases the combined description length of the rule set and the misclassified instances by more than a constant. The rationale for this stopping criterion is that rules that are complex and have many exceptions (misclassified instances) make little sense. Finally, the rules are optimized for compactness.

We used JRip, Weka implementation of RIPPER, with default parameter values.

5 Epitope prediction performance measures

Classification accuracy: The accuracy is defined as the number of correctly classified instances divided by the total number of instances. It is an appropriate measure when the test set has roughly the same number of instances belonging to each class, when misclassifying any class to any other class is equally undesirable and when one is interested in crisp classification. Since we had three times more non-binding than binding peptides, the first condition was not truly satisfied, but we were in addition interested in the classification performance under the assumption that the probability for a binding peptide is the same as for a non-binding one. The other two conditions seem reasonable

for epitope prediction. An advantage of the accuracy is that it is intuitively understandable.

Area under receiver operational characteristics curve: The receiver operational characteristics (ROC) curve is a plot of the true positive rate vs. false positive rate, obtained by varying the threshold above which the value returned by the classifier is considered to indicate the positive class. Examples can be seen in Figures 2 and 8 of the main text. True positive rate (which equals sensitivity) is the number of correctly classified positive instances, divided by the number of all positive instances (the probability to recognize peptides that contain epitopes as such). False positive rate (which equals 1 – specificity) is the number of incorrectly classified negative instances, divided by the number of all negative instances (the probability to mistake a peptide without an epitope for one with an epitope). If true positive rate is larger than false positive rate (the ROC curve lies in the upper left part of the plot), the classification is useful, otherwise it is misleading. The area under the ROC curve (AUC) is an aggregate measure of the performance of the classifier when one considers different thresholds in order to correctly classify more positive instances at the expense of misclassifying negative ones and vice versa. It is appropriate regardless of how many instances belong to each class. Note that binding peptides were considered positive and non-binding negative.

6 EAR rules

The rules describing what distinguishes binding peptides from non-binding ones have the following form:

IF ($a_1 < val_1$) AND ($a_2 \geq val_2$) ... THEN *class* = binding (app_1 / $corr_1$)
...
ELSE IF ($a_{n-1} < val_{n-1}$) AND ($a_n \geq val_n$) ... THEN *class* = binding (app_{m-1} / $corr_{m-1}$)
ELSE *class* = non-binding (app_m / $corr_m$)

The letters a_i indicate the attributes and val_i the values these attributes may take. Some of the attributes are the frequencies of amino acids – these are written as capital letters (for example *Y* for the frequency of tyrosine). The other attributes are amino acid properties (for example *aromaticity*) – these are described in Attributes for Machine Learning. The number *n* is the total number of attribute comparisons and *m* the total number of rules. Each rule applies to a certain percentage of peptides, which is denoted app_i. The rule classifies $corr_i$ of the peptides it applies to correctly.

The percentages of binding peptides that were classified correctly by the rules containing an attribute, which are used in Tables 5 and 7 of the main text to measure the importance of the attribute, were derived from the values app_i and $corr_i$. For a single rule, the value $a_i = app_i \times corr_i$ is the percentage of peptides that were classified correctly by that rule. For all but the last rule, these peptides are binding, so the value $b_i = a_i \times allCount / posCount$ is the percentage of binding peptides that were classified correctly by that rule. The

values *allCount* and *posCount* are the numbers of all the peptides and the binding peptides in a data set. For the complete training set, these numbers are 13,638 and 3,420, respectively; for the classifiable peptides, they are 10,922 and 2,699; for the unclassifiable peptides, they are 2,716 and 721. Summing b_i over all the rules i that contain a given attribute finally yields the percentage of binding peptides that were classified correctly by the rules containing that attribute.

Rules for the whole training set (the summary is in Table 5 of the main text)

IF ($Y \geq 2$) AND (*aromaticity* ≥ 0.2) AND (*polarity* ≤ 0.441152) THEN *class* = binding (5.6 % / 79.3 %)

ELSE IF ($R \geq 1$) AND ($Y \geq 1$) AND (*acidity* ≥ 0.470084) AND (*aromaticity* ≥ 0.095238) AND (*hydrophobicity* ≤ 0.580333) THEN *class* = binding (3.0 % / 71.3 %)

ELSE IF ($R \geq 2$) AND (*aromaticity* ≥ 0.095238) AND (*hydrophobicity* ≤ 0.563667) AND (*preference_for_β-sheets* ≤ 0.461789) THEN *class* = binding (1.5 % / 70.6 %)

ELSE IF (*aromaticity* ≥ 0.266667) AND (*factor_2* ≥ 0.36834) AND (*hydrophobicity* ≤ 0.580333) THEN *class* = binding (3.8 % / 64.7 %)

ELSE IF (*aromaticity* ≥ 0.333333) THEN *class* = binding (1.2 % / 57.9 %)

ELSE IF ($R \geq 1$) AND (*aromaticity* ≥ 0.095238) AND (*polarity* ≤ 0.41893) AND (*factor_2* ≥ 0.380028) THEN *class* = binding (3.1 % / 56.8 %)

ELSE IF (*acidity* ≥ 0.466477) AND (*aromaticity* ≥ 0.133333) AND (*factor_5* ≥ 0.009676) AND (*polarity* ≤ 0.455967) THEN *class* = binding (1.3 % / 56.7 %)

ELSE *class* = non-binding (80.5 % / 85.3 %)

Rules for the classifiable peptides (the summary is in Table 7 of the main text)

IF ($R \geq 1$) AND (*aromaticity* ≥ 0.266667) AND (*polarity* ≤ 0.442798) THEN *class* = binding (4.1 % / 99.3 %)

ELSE IF ($Y \geq 2$) AND (*aromaticity* ≥ 0.2) AND (*polarity* ≤ 0.455967) THEN *class* = binding (4.2 % / 94.1 %)

ELSE IF ($R \geq 2$) AND (*aromaticity* ≥ 0.133333) AND (*factor_5* ≥ 0.008946) AND (*polarity* ≤ 0.455967) THEN *class* = binding (2.7 % / 93.7 %)

ELSE IF (*antigenicity* ≥ 0.423899) AND (*aromaticity* ≥ 0.2) AND (*polarity* ≤ 0.413169) THEN *class* = binding (2.0 % / 92.0 %)

ELSE IF ($R \geq 1$) AND (*aromaticity* ≥ 0.133333) AND (*polarity* ≤ 0.413169) THEN *class* = binding (2.8 % / 77.5 %)

ELSE IF (*aromaticity* ≥ 0.266667) THEN *class* = binding (3.8 % / 71.8 %)

ELSE IF ($H \leq 0$) AND (*aromaticity* ≥ 0.2) AND (*aromaticity* ≤ 0.2) AND (*polarity* ≤ 0.479012) THEN *class* = binding (1.2 % / 75.9 %)

ELSE IF ($Y \geq 1$) AND (*aromaticity* ≥ 0.133333) AND (*factor_5* ≥ 0.008987) AND (*hydrophobicity* ≤ 0.591333) THEN *class* = binding (2.0 % / 58.8 %)

ELSE *class* = non-binding (77.2 % / 93.0 %)

Rules for the unclassifiable peptides (the summary is in Table 7 of the main text)

IF ($R \leq 1$) AND (*aromaticity* ≤ 0.095238) AND (*polarity* ≥ 0.428807) THEN *class* = binding (8.4 % / 13.5 %)

ELSE IF ($A \geq 1$) AND (*antigenicity* ≤ 0.372746) AND (*aromaticity* ≤ 0.153846) AND (*factor_5* ≤ 0.009019) THEN *class* = binding (3.2 % / 73.2 %)

ELSE IF ($R \leq 0$) AND ($Y \leq 0$) AND (*factor_5* ≤ 0.008105) AND (*hydrophobicity* ≥ 0.53) THEN *class* = binding (2.1 % / 82.5 %)

ELSE IF ($C \leq 0$) AND ($Y \leq 0$) AND (*aromaticity* ≤ 0.153846) AND (*occurrence_in_turns* ≤ 0.462481) AND (*preference_for_reverse_turns* ≥ 0.350202) THEN *class* = binding (4.3 % / 64.7 %)

ELSE IF (*aromaticity* ≤ 0) THEN *class* = binding (3.4 % / 52.2 %)

ELSE *class* = non-binding (78.6 % / 86.4 %)

References

1. Karplus PA, Schulz GE (1985) Prediction of chain flexibility in proteins: A tool for the selection of peptide antigens. Naturwissenschaften 72(4): 212-213.
2. Emini EA, Hughes JV, Perlow DS, Boger J (1985) Induction of hepatitis A virus-neutralizing antibody by a virus-specific synthetic peptide. J Virol 55(3): 836–839.
3. Parker JM, Guo D, Hodges RS (1986) New hydrophilicity scale derived from high-performance liquid chromatography peptide retention data: correlation of predicted surface residues with antigenicity and X-ray-derived accessible sites. Biochemistry 25(19): 5425-5432.
4. Kolaska AS, Tongaonkar PC (1990) A semi-empirical method for prediction of antigenic determinants on protein antigens. FEBS Lett 276(1-2): 172-174.
5. Pellequer JL, Westhof E, van Regenmortel MH (1993) Correlation between the location of antigenic sites and the prediction of turns in proteins. Immunol Lett 36(1): 83-99.
6. Blythe MJ, Flower DR (2005) Benchmarking B cell epitope prediction: Underperformance of existing methods. Protein Science 14(1): 246-248.
7. Pellequer JL, Westhof E (1993) PREDITOP: a program for antigenicity prediction. J Mol Graph 11(3): 191-192.
8. Alix AJ (1999) Predictive estimation of protein linear epitopes by using the program PEOPLE. Vaccine 18: 311-314.
9. Odorico M, Pellequer JL (2003) BEPITOPE: predicting the location of continuous epitopes and patterns in proteins. J Mol Recognit 16(1): 20-22.

10. Saha S, Raghava GP (2004) BcePred: Prediction of Continuous B-Cell Epitopes in Antigenic Sequences Using Physico-chemical Properties. Lecture Notes in Computer Science,. pp. 197-204.
11. Rubinstein ND, Mayrose I, Halperin D, Yekutieli D, Gershoni JM, et al. (2008) Computational characterization of B-cell epitopes. Molecular immunology 45: 3477-3489.
12. Westhof E, Altschuh D, Moras D, Bloomer AC, Mondragon A, et al. (1984) Correlation between segmental mobility and the location of antigenic determinants in proteins. Nature 311: 123-126.
13. Emini EA, Hughes JV, Perlow DS, Boger J (1985) Induction of hepatitis A virus-neutralizing antibody by a virus-specific synthetic peptide. Journal of virology 55: 836-839.
14. Parker JM, Guo D, Hodges RS (1986) New hydrophilicity scale derived from high-performance liquid chromatography peptide retention data: correlation of predicted surface residues with antigenicity and X-ray-derived accessible sites. Biochemistry 25: 5425-5432.
15. Pellequer JL, Westhof E, Van Regenmortel MH (1993) Correlation between the location of antigenic sites and the prediction of turns in proteins. Immunology letters 36: 83-99.
16. El-Manzalawy Y, Dobbs D, Honavar V (2008) Predicting linear B-cell epitopes using string kernels. Journal of molecular recognition : JMR 21: 243-255.
17. El-Manzalawy Y, Dobbs D, Honavar V (2008) Predicting flexible length linear B-cell epitopes. Computational systems bioinformatics / Life Sciences Society Computational Systems Bioinformatics Conference 7: 121-132.
18. Taylor WR (1986) The classification of amino acid conservation. J Theor Biol 119(2): 205-218.
19. Janin J, Wodak S (1978) Conformation of amino acid side-chains in proteins. J Mol Biol 125(3): 357-386.
20. Bordo D, Argos P (1991) Suggestions for safe residue substitutions in site-directed mutagenesis. J Mol Biol 217: 721-729.
21. Grantham R (1974) Amino acid difference formula to help explain protein evoulution. Science 185(4154): 862-864.
22. Smith DK, Radivojac P, Obradovic Z, Dunker AK, Zhu G (2003) Improved amino acid flexibility parameters. Protein Science 12(5): 1060-1072.
23. Berg JM, Tymoczko JL, Strye L (2002) Biochemstry - 5th Edition. New York: W H Freeman.
24. Levitt M (1978) Conformational preferences of amino acids in globular proteins. Biochemistry 17(20): 4277-4285.
25. Atchley WR, Zhao J, Fernandes AD, Drüke T (2005) Solving the protein sequence metric problem. Proc Natl Acad Sci U S A 102(18): 6395-6400.
26. Hall M, Frank E, Holmes G, Pfahringer B, Reutemann P, et al. (2009) The WEKA data mining software: an update. ACM SIGKDD Explorations Newsletter 11(1): 10-18.
27. Platt J (1998) Fast Training of Support Vector Machines Using Sequential Minimal Optimization. In: Schölkopf B, Burges C, Smola AJ, editors. Advances in Kernel Methods: Support Vector Learning: The MIT Press. pp. 185-208.

28. le Cessie S, van Houwelingen JC (1992) Ridge estimators in logistic regression. Appl Statist 41(1): 191-201.
29. Frank E, Wang Y, Inglis S, Holmes G, Witten IH (1998) Using Model Trees for Classification. Machine Learning 32: 63-76.
30. Wang H, Witten IH (1999) Pace regression. Working paper: 1-27.
31. Üstün B, Melssen WJ, Buydens LMC (2006) Facilitating the application of Support Vector Regression by using a universal Pearson VII function based kernel. Chemometrics and Intelligent Laboratory Systems 81(1): 29-40.
32. Platt J (1999) Fast training of support vector machines using sequential minimal optimization. In: Smola AH, Bartlett P, Schölkopf B, Schuurmans E, editors. Advances in Large Margin Classifiers: MIT Press. pp. 61-74.
33. Wolpert DH (1992) Stacked Generalization. Neural Networks 5: 241-259.
34. Seewald A. How to Make Stacking Better and Faster While Also Taking Care of an Unknown Weakness; 2002.
35. Cohen WW. Fast effective rule induction; 1995. pp. 115-123.
