# Supplementary material for: Epitope Predictions Indicate the Presence of Two Distinct Types of Epitope-Antibody-Reactivities Determined by Epitope Profiling of Intravenous Immunoglobulins
Source: PLoS One. 2013 Nov 11;8(11):e78605. doi: 10.1371/journal.pone.0078605 (PMC3823795; doi:10.1371/journal.pone.0078605)
Supplement: File S4 — (1) Additional machine learning results: Confusion matrices of dividing peptides into classifiable and unclassifiable. (2) Additional PWM results: heat maps for the 1st degree classifiable and unclassifiable peptides, respectively; ROC curve for 2nd degree unclassifiable peptides, now classified by PWM scores. (3) Details on MHC class I and class II prediction servers. (PDF) [file pone.0078605.s008.pdf]

Supporting information File S4

1 Additional Machine Learning Results

File S4 Table 1 shows confusion matrices of dividing peptides into 1st degree classifiable and unclassifiable, which complement Table 5 of the main text.

**Input dataset**	**Prediction**	**Actual binders**	**Actual non-binders**
Whole training set	Binding	1,789	694
	Non-binding	1,631	9,524
1st degree classifiable	Binding	2,609	46
	Non-binding	90	8,177
1st degree unclassifiable	Binding	566	77
	Non-binding	155	1,918

File S4 Table 1. Confusion matrices of dividing peptides into 1st degree classifiable and unclassifiable. Number of respective predicted peptides when the classifier was 10-fold cross-validated on all the peptides in the original training set, or on peptides that the first one classified correctly (1st degree classifiable) or incorrectly (1st degree unclassifiable), respectively. All classifiers used the interpretable attributes and logistic regression. The numbers of correct predictions are underlined.

2. Additional Position Weight Matrix (PWM) Results

File S4 Figures 1 and 2 show the ratio PWM (binding versus non-binding peptides) of the 1st degree classifiable and unclassifiable peptides, respectively. These data were used to construct the heatmaps depicted in File S4 Figures 3 and 4. The colors clearly show the difference between the two groups: in File S4 Figure 3, the heat map for the 1st degree classifiable peptides, the amino acids Y, W, H, R, M, C and F show relatively strong pink saturation indicating stronger representation in positive peptids while the rest of the amino acids are labeled in cyan hues; in File S4 Figure 4, visualizing the result for 1st degree unclassifiable peptides, the opposite is the case. File S4 Figure 5shows the ratio PWM of the 2nd degree unclassifiable peptides depicted in Figure 6 of the main manuscript as a heat map.

	Positions within peptide														
Amino acid	P1	P2	P3	P4	P5	P6	P7	P8	P9	P10	P11	P12	P13	P14	P15
A	0.641407	0.739982	0.643399	0.716867	0.659433	0.738275	0.603847	0.728175	0.654357	0.715308	0.639087	0.591985	0.664911	0.590399	0.608251
C	1.584276	1.425062	2.495379	1.971384	1.833403	2.704709	1.866437	2.341136	2.191474	2.351245	2.611443	1.591961	2.064975	1.708421	1.81683
D	0.851383	0.771512	0.83692	0.832906	0.749067	0.842232	0.771614	0.677041	0.67457	0.605301	0.745232	0.617302	0.782634	0.896622	0.806226
E	0.48648	0.533726	0.50107	0.443312	0.463917	0.472529	0.509514	0.386323	0.363209	0.347608	0.333825	0.389426	0.420232	0.440597	0.380835
F	2.499464	3.030215	2.05857	2.82156	2.657746	2.437347	2.938518	3.046684	3.132265	2.852215	3.270452	3.192536	3.18517	3.531825	4.096555
G	1.003752	1.081081	0.952824	0.80696	0.930285	0.812449	1.01758	1.034253	0.984057	1.096132	1.267792	0.976122	0.985536	1.31167	0.968436
H	1.203812	1.406162	1.229134	1.748097	1.388721	1.378262	1.896611	1.395578	1.465857	1.986236	1.998883	2.163838	1.836757	1.866754	2.449295
I	1.350331	1.136461	0.989588	0.950126	1.249712	0.976501	0.895613	0.863227	0.989183	0.944472	0.807173	0.790687	0.757765	0.886601	0.886465
K	0.804886	0.770723	0.738753	0.825483	0.952089	0.82988	1.00722	0.724212	0.853524	0.919111	0.941061	0.928242	0.77327	0.808906	0.815494
L	1.017029	1.304299	1.270295	1.114998	0.994181	1.181567	1.142506	1.074704	1.048626	0.985155	1.080188	0.974204	1.137241	1.300212	1.37576
M	2.047503	1.523342	1.564963	1.531154	1.592585	1.919828	2.320167	1.545104	1.938799	1.604371	1.396397	1.774245	1.71376	3.163864	2.820071
N	0.843055	0.842048	0.884239	0.787936	0.660365	0.633771	0.782134	0.839206	0.656334	0.661296	0.588921	0.769689	0.657896	0.795176	0.558422
P	0.820693	0.662983	0.842581	0.829836	0.717175	0.621265	0.72034	0.508702	0.792677	0.678237	0.837425	0.829836	0.943169	0.796361	0.869587
Q	0.626623	0.662905	0.585167	0.53146	0.802129	0.685334	0.724386	0.435241	0.53555	0.510935	0.45281	0.493273	0.407	0.466609	0.503365
R	1.329312	1.32077	1.601865	1.427635	1.310866	1.395806	1.700475	1.162466	1.977374	1.89291	2.060992	1.80427	1.920334	2.031123	2.050842
S	0.638071	0.568934	0.669601	0.664346	0.633232	0.710457	0.481364	1.069288	0.669285	0.570993	0.516752	0.660528	0.657376	0.640023	0.582142
T	0.961427	0.681818	0.804426	0.921403	0.862269	0.818324	0.676136	1.184505	0.713187	0.801068	0.644832	0.764706	0.598156	0.889191	0.736112
V	0.983011	0.929832	0.981989	1.026251	0.967528	0.919309	0.981482	0.698476	0.790103	0.791025	0.872236	0.860887	0.720241	0.721766	0.939567
W	2.917038	3.864087	2.792794	2.711884	3.503687	3.523875	3.43234	1.821729	3.808355	4.648146	4.415484	5.271247	5.063503	7.494843	9.749389
Y	3.692168	4.113023	4.195872	5.716459	5.416327	4.911465	4.28712	8.202611	5.386102	5.906291	5.279168	5.131257	5.82375	6.808406	10.24794

File S4 Figure 1: Ratio PWM of amino acids in 1st degree classifiable peptides that were "binding" versus those that were "not-binding" to IVIG antibodies

Each component of the PWM corresponds to the ratio of the frequency of the occurrence of a given amino acid (row) at a given peptide position (column) in "binding" vs. "non-binding" peptides. Green shading represent amino acids that are more more abundant in "binding" peptides than in "non-binding" ones (threshold >2). In contrast red shading label amino acids that are more more abundant in "non-binding" peptides (threshold < 0.6).

	Positions within peptide														
Amino acid	P1	P2	P3	P4	P5	P6	P7	P8	P9	P10	P11	P12	P13	P14	P15
A	1.077515	1.662896	2.006701	1.352941	1.330317	1.261029	1.058079	1.879795	1.47549	1.484848	1.35835	1.441176	1.347594	0.827606	0.783552
C	0.496957	0.313299	0.418406	0.432353	1	0.375959	0.823529	0.83268	0.598224	0.800654	0.768627	0.706992	0.674593	0.882353	0.366013
D	1.204565	1.017301	1.549265	0.913917	1.398789	1.415893	1.067538	1.281046	1.415893	1.327399	1.20098	1.076078	1.403251	1.10556	1.512054
E	1.522141	1.661592	1.806275	1.990196	1.351103	1.811765	1.391481	2.690196	1.502503	1.703209	1.632176	1.695502	1.935294	1.525952	0.939194
F	0.496957	0.308824	0.296317	0.628877	0.399534	0.453704	0.526584	0.47544	0.627609	0.524064	0.557093	0.598224	0.582014	0.379257	0.689258
G	1.116587	0.906546	0.936149	0.907407	1.057744	0.95243	0.761376	1.195122	1.131391	1.070588	1.053167	0.777778	1.102813	0.858573	1.179144
H	0.989464	0.709502	0.26548	0.852527	0.653333	0.817965	0.555634	0.566176	0.751918	0.760621	0.720588	1.098039	0.720588	0.665158	0.992285
I	1.493267	0.9199	0.864706	0.900735	1.076923	0.868179	1.213622	1.601307	0.810662	0.70383	0.935501	1.159971	0.95197	0.891449	0.408522
K	1.371216	0.98366	1.235294	1.173347	0.968471	1.136312	1.210588	1.179144	0.97944	1.048128	0.85053	1.427184	0.934817	1.080882	0.971228
L	0.830796	0.876505	0.870711	0.592051	0.828262	1.119031	1.059178	0.967365	0.90184	0.925937	0.966338	0.821135	0.812155	1.048128	0.954665
M	0.683445	0.870144	0.535294	1.142064	0.517345	0.691765	0.688323	0.681283	0.771334	0.52726	0.690141	0.730196	0.879362	0.745436	0.816667
N	1.52852	1.773756	1.464052	1.134041	1.221336	1.419005	0.864706	0.890138	1.191373	1.39614	1.348197	1.215993	1.124821	1.026591	0.976281
P	1.25641	0.861734	1.421434	1.274303	0.832019	1.250832	1.120915	1.036352	0.836812	0.867257	1.61693	1.298643	1.422934	1.136703	1.048128
Q	2.087221	1.580645	1.904412	2.656286	1.875817	1.367896	2.130435	1.306066	1.653114	1.419666	1.365325	1.394687	1.370299	1.129571	1.393137
R	0.788568	0.607996	0.650854	0.783798	0.830508	0.740421	0.941759	0.32412	0.661524	0.890483	0.737882	0.755568	0.839741	0.684916	0.634647
S	1.196448	1.683751	1.390609	1.552036	1.635181	1.367896	1.213622	1.508732	1.673624	1.537255	1.705882	1.246423	0.969067	1.138408	1.173347
T	1.044331	1.909559	1.258492	1.20098	1.380282	1.193702	1.183824	1.294616	0.823529	1.120915	1.076541	1.110907	1.313072	1.136703	1.080882
V	1.095294	1.337174	1.053763	0.951176	1.441176	1.066471	1.129169	1.45854	1.642271	1.023639	0.870711	0.581315	1.127877	0.6939	0.609729
W	0.097707	0.320261	0.294118	0.194754	0.288235	0.508651	0.256209	0.137255	0.665158	0.804378	0.536252	1.048128	0.598224	0.435072	2.058824
Y	0.13992	0.205882	0.335808	0.192157	0.134063	0.269379	0.624015	0.27639	0.264706	0.364087	0.31392	0.399534	0.313299	0.270221	0.182427

File S4 Figure 2: Ratio PWM of amino acids in 1st degree unclassifiable peptides that were "binding" versus those that were "not-binding" to IVIG antibodies

Each component of the PWM corresponds to the ratio of the frequency of the occurrence of a given amino acid (row) at a given peptide position (column) in "binding" vs. "non-binding" peptides. Green shading represent amino acids that are more more abundant in "binding" peptides than in "non-binding" ones (threshold >2). In contrast red shading label amino acids that are more more abundant in "non-binding" peptides (threshold < 0.6).

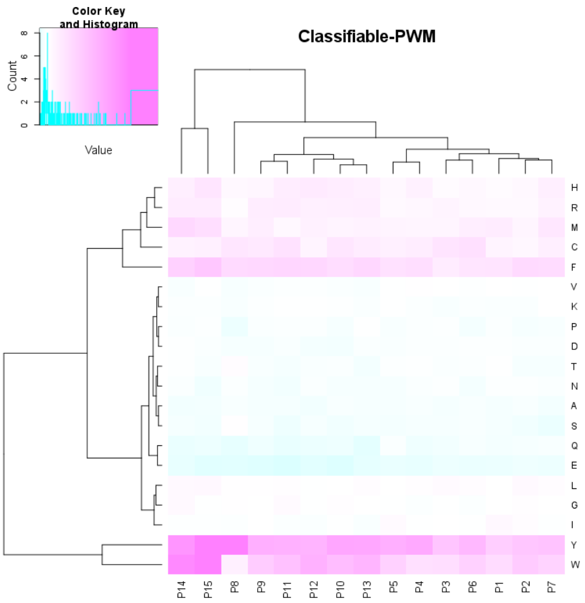

File S4 Figure 3: Heat map for the 1st degree classifiable peptides in the training set. The rows represent the individual amino acids, the columns the positions within the 15mer peptide. The heat map color reflects the ratio between the PMW matrix values for the "binding" and the "non-binding" peptides in the set. Pink color indicates high propensity (overrepresentation in "binding" peptides), while blue color indicates low propensity (underrepresentation in "binding" peptides). Standard hierarchical clustering using Euclidean distance.was performed on rows and columns.

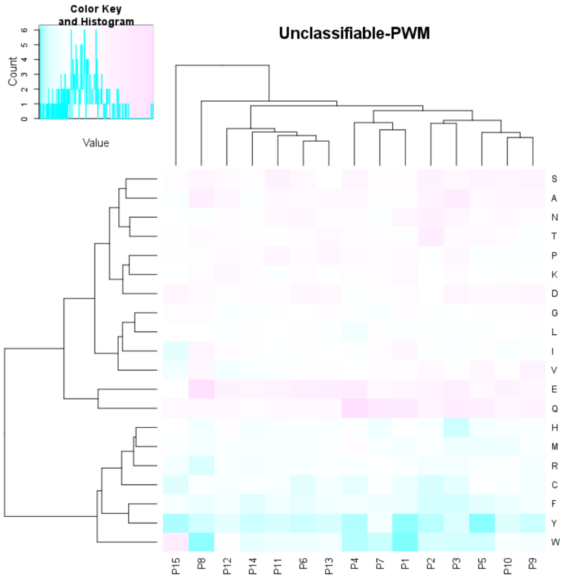

File S4 Figure 4: Heat map for the 1st degree unclassifiable peptides in the training set. The rows represent the individual amino acids, the columns the positions within the 15mer peptide. The heat map color reflects the ratio between the PMW matrix values for the "binding" and the "non-binding" peptides in the set. Pink color indicates high propensity (overrepresentation in "binding" peptides), while blue color indicates low propensity (underrepresentation in "binding" peptides). Standard hierarchical clustering using Euclidean distance.was performed on rows and columns.

	Positions within peptide														
Amino acid	P1	P2	P3	P4	P5	P6	P7	P8	P9	P10	P11	P12	P13	P14	P15
A	2.637681	1.413043	0.741848	1.978261	0.719368	0.304348	1.648551	1.695652	0.879227	1.758454	1.153986	0.152174	1.130435	0.456522	0.741848
C	0.565217	1.978261	0.98913	0.282609	1.483696	0.65942	0.32971	0.98913	0.98913	0	2.967391	1.978261	0.98913	0	3.297101
D	0.741848	0.847826	0.65942	0.65942	1.130435	0.65942	0.879227	1.483696	0.565217	2.373913	0.98913	0.741848	0.593478	0.98913	0
E	1.186957	1.730978	0.539526	1.695652	0.76087	1.438735	1.758454	0.98913	0.847826	0.593478	0.247283	0.359684	0.494565	0.465473	0.456522
F	0.494565	0.65942	0.899209	0.263768	0.494565	1.758454	1.648551	0.219807	1.130435	0.565217	0.791304	9.891304	3.956522	0.98913	1.130435
G	1.055072	1.079051	0.304348	0.98913	0.791304	2.769565	1.055072	1.079051	1.648551	1.438735	1.318841	0.847826	1.099034	0.65942	0.395652
H	1.186957	0	1.483696	1.648551	0.65942	0.98913	0.395652	3.956522	0.65942	0.32971	1.978261	0.98913	2.472826	0.791304	1.978261
I	0.791304	0.98913	2.769565	0.741848	2.472826	1.483696	2.472826	0.593478	0.814578	0.899209	1.384783	1.730978	0.219807	2.967391	0.791304
K	0.923188	0.565217	0.76087	0.899209	1.978261	1.538647	0.76087	0	1.079051	2.373913	0.824275	0.923188	1.055072	0.65942	2.637681
L	1.318841	1.032136	0.899209	0.809289	0.65942	0.777174	1.036232	0.344045	2.119565	1.036232	0.65942	1.055072	1.438735	2.130435	1.079051
M	0.494565	1.695652	0.98913	0.98913	1.130435	0.247283	1.978261	0.65942	0.65942	1.582609	1.318841	1.978261	1.186957	1.483696	1.483696
N	1.978261	1.186957	4.615942	0.565217	0.98913	1.483696	0	1.318841	1.318841	0.395652	1.079051	0.65942	0.98913	0	0.494565
P	1.483696	1.186957	0.456522	0.913043	0.824275	0.98913	0.32971	1.153986	0.539526	1.186957	1.079051	1.318841	0.98913	1.217391	0.98913
Q	0.359684	1.483696	1.258893	1.236413	1.099034	0.847826	0.65942	1.978261	1.130435	0.304348	0.65942	0.899209	0.494565	0.395652	1.384783
R	0.179842	0.719368	0.98913	0.923188	1.186957	1.730978	0.706522	2.517787	1.236413	1.648551	0.824275	0.824275	0.76087	4.615942	1.318841
S	1.618577	1.978261	0.395652	1.521739	1.582609	0.791304	0.565217	0.870435	0.395652	0.98913	0.296739	1.318841	1.130435	0.395652	0.65942
T	0.247283	1.258893	3.956522	1.483696	1.079051	0.247283	1.798419	1.648551	0.899209	0.608696	1.236413	0.65942	1.236413	1.483696	0.494565
V	0.879227	0.69821	2.176087	1.978261	0.539526	0.879227	0.98913	0.527536	1.079051	0.847826	1.271739	0.423913	0.494565	2.769565	1.758454
W	1.582609	0.32971	0.847826	0.98913	0	0	0	0.98913	0	0.32971	0.98913	0	0.395652	0	2.967391
Y	0.791304	0.494565	0.65942	0.98913	1.978261	1.318841	3.956522	1.318841	1.483696	0.741848	3.461957	9.891304	1.483696	1.483696	1.582609

File S4 Figure 5: Ratio PWM of amino acids in 2nd degree unclassifiable peptides that were "binding" versus those that were "not-binding" to IVIG antibodies

Each component of the PWM corresponds to the ratio of the frequency of the occurrence of a given amino acid (row) at a given peptide position (column) in "binding" vs. "non-binding" peptides. Green shading represent amino acids that are more more abundant in "binding" peptides than in "non-binding" ones (threshold >2). In contrast red shading label amino acids that are more more abundant in "non-binding" peptides (threshold < 0.6).

In contrast to the failure of the machine learning approach to further classify the 274 peptides initially found 2nd degree unclassifiable, the PWM-approach shows good performance (File S4 Figure 6) and only a very limited number of peptides were left even 3rd degree unclassifiable (see Figure 1 main manuscript)

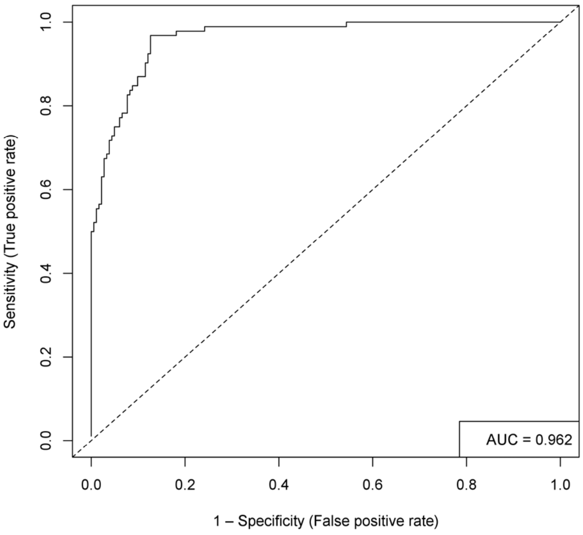

File S4 Figure 6: ROC curve performance chart for the prediction of "binding"\"non-binding" based on PWM scores. The input set consisted of only those peptides that were initially 2nd degree unclassifiable by machine learning ML-simple. For this set another round of machine learning did not lead to succesful further classification.

3. MHC Class I and Class II Prediction Servers

To test how a classifier meant for MHC class II T-cell epitopes predicts the epitopes in our dataset, we used the NetMHCIIpan 1.1 server [1]. The NetMHCIIpan server was found to be the best publicly accessible MHC class II peptide-binding-prediction server in a recent survey [2]. The server predicts binding to more than 500 HLA-DRB1, DRB3, DRB4 and DRB5 alleles (different types of MHC class II molecules). We used it to predict the binding of peptides to the 10 most common HLA-DRB1 alleles in the USA population of European descent [3]: DRB1*1501, DRB1*0701, DRB1*0301, DRB1*0101, DRB1*0401, DRB1*1301, DRB1*1101, DRB1*1302, DRB1*0404, DRB1*1104. In addition, we considered alleles DRB3*0101, DRB4*0101 and DRB5*0101. For each peptide, we used the maximum of the 13 binding affinities as the final prediction.

Furthermore, we used a classifier for MHC class I T-cell epitopes: NetMHC 3.2 [4]. The NetMHC server was found to be the best publicly accessible MHC class I peptide-binding-prediction server in another recent survey [5]. The server predicts binding to 57 HLA-A and B alleles, and some animal alleles (different types of MHC class I molecules). We used it to predict the binding of peptides to the 10 most common HLA-A and B alleles in the USA population of European descent [3]: A*0201, A*0101, A*0301, B*0702, B*0801, B*4402, A*2402, B*1503, B*3501, B*4001. For each peptide, we used the maximum of the 10 binding affinities as the final prediction.

The peptides containing the amino acids Z and X (335 peptides) and the peptides longer than 15 amino acids (5 peptides) were not classified by the MHC servers. These peptides constitute 2.5 % of the training set.

References:

1. Nielsen M, Lundegaard C, Blicher T, Peters B, Sette A, et al. (2008) Quantitative predictions of peptide binding to any HLA-DR molecule of known sequence: NetMHCIIpan. PLoS Comput Biol 4(7): e1000107.
2. Lin HH, Zhang GL, Tongchusak S, Reinherz EL, Brusic V (2008) Evaluation of MHC-II peptide binding prediction servers: applications for vaccine research. BMC Bioinformatics 9 Suppl 12: S22.
3. Maiers M, Gragert L, Klitz W (2007) High-resolution HLA alleles and haplotypes in the United States population. Hum Immunol 68(9): 779-788.
4. Lundegaard C, Lamberth K, Harndahl M, Buus S, Lund O, et al. (2008) NetMHC-3.0: accurate web accessible predictions of human, mouse and monkey MHC class I affinities for peptides of length 8-11. Nucleic Acids Research 36: W509-512.
5. Lin HH, Ray S, Tongchusak S, Reinherz EL, Brusic V (2008) Evaluation of MHC class I peptide binding prediction servers: applications for vaccine research. BMC immunology 9: 8.
